# The Overview of Silicon Carbide Technology: Status, Challenges, Key Drivers, and Product Roadmap

**DOI:** 10.3390/ma18010012

**Published:** 2024-12-24

**Authors:** Maciej Kamiński, Krystian Król, Norbert Kwietniewski, Marcin Myśliwiec, Mariusz Sochacki, Bartłomiej Stonio, Ryszard Kisiel, Agnieszka Martychowiec, Katarzyna Racka-Szmidt, Aleksander Werbowy, Jarosław Żelazko, Piotr Niedzielski, Jan Szmidt, Andrzej Strójwąs

**Affiliations:** 1 Institute of Microelectronics and Optoelectronics, Warsaw University of Technology, 75 Koszykowa Str., 00-662 Warsaw, Poland; maciej.kaminski@pw.edu.pl (M.K.); krystian.krol@pw.edu.pl (K.K.); norbert.kwietniewski@pw.edu.pl (N.K.); marcin.mysliwiec@pw.edu.pl (M.M.); bartlomiej.stonio@pw.edu.pl (B.S.); ryszard.kisiel@pw.edu.pl (R.K.); agnieszka.martychowiec@pw.edu.pl (A.M.); aleksander.werbowy@pw.edu.pl (A.W.); jan.szmidt@pw.edu.pl (J.S.); 2 Łukasiewicz Research Network—Institute of Microelectronics and Photonics, 32/46 Al. Lotników, 02-668 Warsaw, Poland; katarzyna.racka@imif.lukasiewicz.gov.pl (K.R.-S.); jaroslaw.zelazko@imif.lukasiewicz.gov.pl (J.Ż.); 3 Center for Advanced Materials and Technology CEZAMAT, Warsaw University of Technology, 19 Poleczki Str., 02-822 Warsaw, Poland; 4 Institute of Materials Science and Engineering, Lodz University of Technology, 1/15 Stefanowskiego Str., 90-537 Łódź, Poland; piotr.niedzielski@p.lodz.pl; 5 Carnegie Mellon University, 5000 Forbes Avenue, Pittsburgh, PA 15213, USA; ajs@ece.cmu.edu; 6 PDF Solutions, Inc., 2858 De La Cruz Blvd, Santa Clara, CA 95050, USA

**Keywords:** SiC MOSFET, SiC technology, automotive industry, battery electric vehicles

## Abstract

Arguably, SiC technology is the most rapidly expanding IC manufacturing technology driven mostly by the aggressive roadmap for battery electric vehicle penetration and also industrial high-voltage/high-power applications. This paper provides a comprehensive overview of the state of the art of SiC technology focusing on the challenges starting from the difficult and lengthy SiC substrate growth all the way to the complex MOSFET assembly processes. We focus on the differentiation from the established Si manufacturing processes and provide a comprehensive list of references as well as a brief description of our own research into the key manufacturing processes in this technology. We also present a SiC technology and product roadmap.

## 1. Introduction

### 1.1. SiC Technology Market and Product Trends

Without any doubt, SiC is a key emerging technology for the next generation of semiconductors driving the electromobility, renewable energies, smart grids, smart buildings, smart metering, and digitization of industrial processes leading to energy transformation. SiC holds great promise for several automotive and traction applications, particularly for battery electric vehicles (BEVs) with charging systems, and has already been widely adopted. Moreover, it plays a crucial role in high-power industrial and public transport applications.

The main reason is the material itself since, compared to Si, it offers the following:

- 10× higher maximum electric field;
- 2× higher electron saturation velocity;
- 3× higher energy bandgap;
- 3× higher thermal conductivity.

As a result, SiC MOSFETs are characterized by extremely low switching power losses, are faster and more robust than silicon, and have a smaller die size for equivalent breakdown voltage.

Moreover, SiC-based products can operate at higher frequencies which makes them ideal for the 5G systems, and they have lower power losses and allow for the design of smaller, lighter, and more energy-efficient systems. They can even operate far above 200 °C junction temperatures which allows for the reduction in cooling requirements and, importantly, increased lifetime. Compared to GaN devices, SiC MOSFETs can operate at much higher voltages and therefore power levels.

With the benefits mentioned above, SiC’s advantage increases in parallel with rising operating voltage. Relative to silicon, 1200 V SiC switches have added value compared to 600 V switches. These properties have driven a radical transformation in SiC power switching devices, substantially improving system efficiency in EVs and EV charging as well as energy infrastructure, making SiC an ideal choice for automakers and public transportation worldwide.

Electrical vehicles are experiencing a massive boom fueled by the governmental regulations imposing CO_2_ limits that drive the demand for SiC. It is estimated that by 2050, the demand for SiC wafers will be comparable to the current number of 300 mm Si wafers produced by the world’s largest foundry. Of course, SiC MOSFET fabrication processes are more complicated than Si, especially the substrate growth, but there is an intense race among semiconductor companies to satisfy this exponentially growing demand.

The SiC ecosystem has been radically changed in recent years due to the vertical integration of wafer manufacturing and module packaging. While Wolfspeed is still a dominant SiC wafer supplier, companies like ST, Infineon, ROHM, and Onsemi are quickly becoming vertically integrated producers from the SiC wafers to the final product. Another trend is the transition from the 150 mm (6 inch) to the 200 mm (8 inch) wafer sizes which allows for cost reduction in SiC MOSFET manufacturing [1].

Following Tesla’s adoption of SiC in its main inverter in 2017, automotive use has become the killer application for SiC. Since then, we have witnessed an interest in SiC from almost all carmakers and Tier 1 suppliers [2]. The dominance of automotive applications is even more pronounced: from 63% to 80%.

Along with EV applications, there is a trend to adopt SiC in charging infrastructure, where it offers increased efficiency and reduced system size. In addition, SiC is forecast to grow at a double-digit compound annual growth rate between 2019 and 2027 in applications such as rail, motor drives, and photovoltaics.

### 1.2. Paper Overview

In addition to the challenges of producing high-quality epitaxial structures with a sufficiently large diameter, fundamental differences in the manufacturing process between mature silicon technology and emerging silicon carbide technology for doping active layers, defining high-aspect-ratio shapes, producing the appropriate SiO_2_/semiconductor interface quality, and fabricating interconnections have slowed the implementation of the next generation of power devices for many years. To meet the global market and product trends described in the previous section, our group began basic research in this field in the late 1990s. The main focus of this paper is to review the current status and the main challenges of SiC technology and describe the challenges that are quite different from Si manufacturing processes. We provide a comprehensive review of the state of the art of technological advances and also a brief description of the contributions of the research groups at the Warsaw University of Technology as well as affiliated Polish research institutes. We start with the description of the SiC MOSFET process flow starting from the complex and lengthy SiC substrate growth to the packaging of the power MOSFET. Section 3 is devoted to the ion implementation in SiC which is carried out at much higher temperatures than in Si fabrication. The defectivity issues are also addressed in this section. Section 4 provides a brief overview of the dry etching technology choices and challenges. The next section is devoted to the thermal oxidation issues in SiC technology and a lot of attention is paid to the factors affecting the oxide quality. Section 6 presents the challenges of contact creation in SiC technology. The following section is devoted to the complex assembly processes for high-power SiC products. Finally, we conclude the paper by summarizing the SiC technology readiness for massive expansion in the foreseeable future.

## 2. SiC MOSFET Process Flow

**Figure 1:**
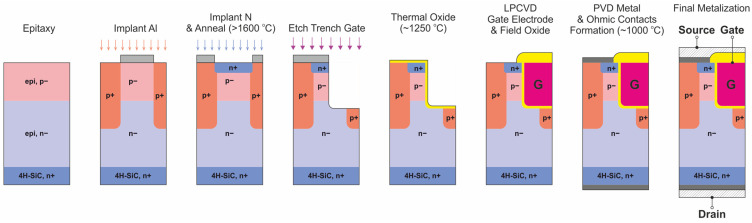
An example of the SiC MOSFET manufacturing process flow.

The device manufacturing process determines the final parameters of SiC power MOSFETs (blocking voltage, on-resistance, gate charge, threshold voltage). The processing will primarily affect the efficiency of dopant electrical activation, channel mobility, recombination lifetime, specific contact resistance, which determines on-resistance, and many other on-state, off-state, and switching effects. A typical process flow is presented in Figure 1. Other examples of the SiC power transistor manufacturing process can also be found in the latest references [3]. Due to the performance of power diodes and transistors, and due to the general trend of increasing the density of individual devices on the substrate wafer, the construction of devices using the trench structure, well known from silicon technology, has become attractive in the last decade for SiC MOSFET manufacturing.

The growth of the SiC substrate is a challenging process that requires strict control to produce high-quality low-defectivity material. The most successful growth method for bulk SiC single crystals is the sublimation process known as physical vapor transport (PVT) which consists of the following procedures: (1) sublimation of SiC source, (2) mass transport of sublimed species, and (3) surface reaction and crystallization.

**Figure 2:**
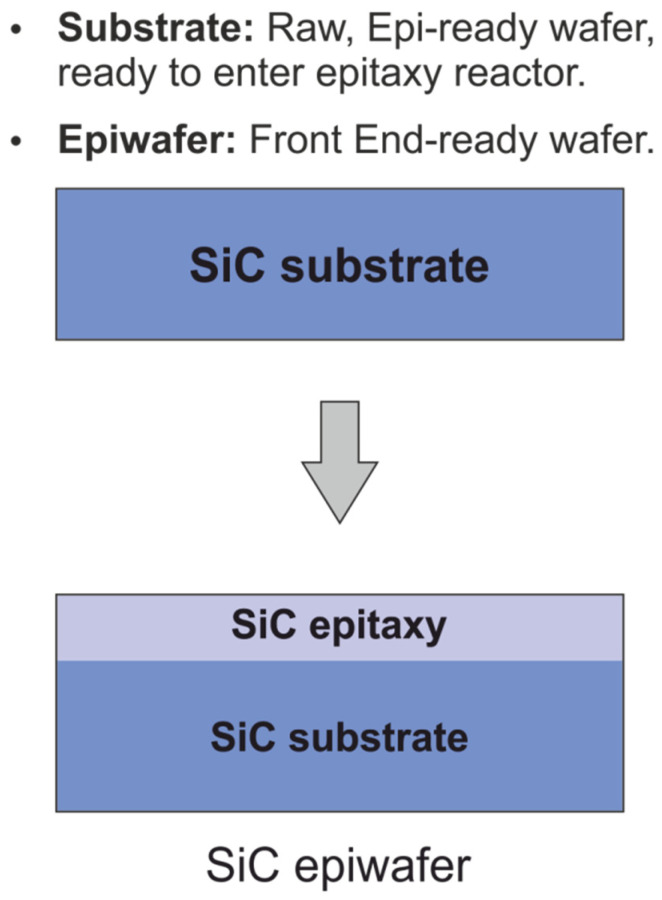
Epitaxial layer growth.

The growth rate is low, and it takes several days to produce the SiC boule which is then cut into wafers on which the epitaxial layers are grown as shown in Figure 2 [4]. The starting substrate is a highly doped (n+) 4H-SiC wafer, playing the role of the transistor’s drain afterwards. Subsequently, onto this substrate, low-doped ntype (n−) and then p-type (p−) epitaxial layers are grown. Prior to the epitaxy process, the surface of the substrate is cleaned in a chemical bath and then, in the epitaxy reactor, is etched into hydrogen to reduce the number of defects that otherwise might have formed in the growing epitaxial film.

**Figure 3:**
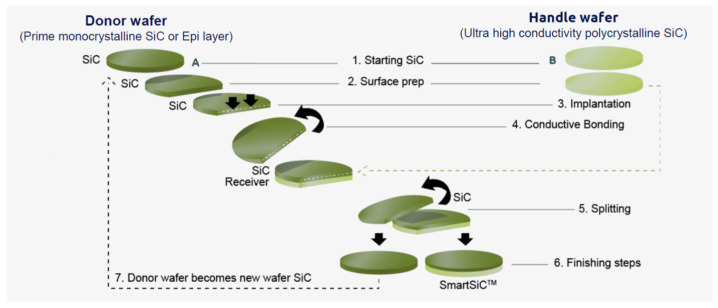
SOITEC’s Smart Cut SiC wafer manufacturing process. Reproduced with permission from [5].

Recently, SOITEC proposed a novel process for producing SiC wafers using their Smart Cut process shown in Figure 3 [5].

The donor wafer is the prime monocrystalline SiC substrate wafer obtained from the bulk wafer growth process described above and the handle wafer is the high conductivity polycrystalline wafer which allows for higher current densities, i.e., smaller dies and hence more die per wafer. Moreover, it results in better flatness, lower surface defectivity (higher yield), and also a simplified backside ohmic contact process which does not require annealing, thus reducing the number of process steps. As can be seen in the figure, the donor wafer may be re-used several (more than 10) times which is important as the substrate manufacturers cannot keep up with the demand. This technique has definitely upgraded the SiC wafer manufacturing process and has already been adopted by part of the SiC producers.

The next step in defining the SiC transistor’s topography is the mask preparation for the p+ region implantation. The role of this mask is to prevent ions being implanted from reaching the epitaxial layer, except for the well-defined windows that are opened in the photolithography process. This implantation mask is often an Al layer. The depth of the implanted p+ regions has to be larger than the thickness of the p− epitaxial layer so as to form an effective grounding or shielding region of the MOSFET body. For p+ implantation, Al ions are used. Implantation of the transistor’s n+ source regions consists of analogous technological steps (formation of Al implantation mask and windows opening in the photolithographic process), with the only difference being that, in this case, N ions are implanted and it is performed in a much shallower manner. The market availability of production-grade n-type wafers puts a lot of emphasis on the development of p-type doping processes, which are typically carried out by a high-temperature aluminum ion implantation technique with post-implantation annealing aimed mainly at improving the level of electrical activation of the implanted dopant. Nitrogen is a commonly used n-type dopant for SiC. In addition to defining active regions in a semiconductor device with a specific dopant distribution, ion implantation is commonly used to manufacture electric field-modulating regions at the edge of the device (junction termination extension) that prevent premature electrical breakdown. Due to the significant importance of the described processes of doping and electrical activation, a separate, detailed section has been devoted to these issues.

After the implantation process and Al mask removal, the substrate surface is cleaned and then covered with a protective carbon (graphite) cap. Subsequently, it is annealed at a high temperature (1600–1700 °C) to activate implanted dopant atoms as well as to recrystallize material structure amorphized during the ion bombardment.

The next step is the formation of trenches for transistor gates via the deep etching of SiC through the hard mask in the inductively coupled plasma (ICP) process. The implementation of the trench-type structure requires the development of a specialized dry etching process for SiC epitaxial structures. The goal is to obtain extremely low values of the on-resistance of power devices while maintaining the highest long-term stability. The inductively coupled plasma–reactive ion etching (ICP-RIE) process is widely used for the manufacturing of trench structures, which is described in a separate section of this review paper, including the presentation of our own research results.

After fabricating the trenches, the substrate is again cleaned and then oxidized at a high temperature to produce gate insulation. Oxidation is usually carried out in a N_2_O, NO, or dry O_2_ atmosphere at temperatures around 1000–1100 °C. The critical issue in the commercialization of advanced semiconductor devices in SiC technology was the insufficient quality of the gate dielectric to ensure an extremely low near-interface trap density while maintaining the required reliability and long-term stability of the interface and dielectric. Therefore, we decided to prepare an up-to-date review of the literature devoted to this subject, also including our own results, mainly concerning the kinetics of the thermal oxidation of silicon carbide. After the gate dielectric formation, the polysilicon gate contacts as well as the gate-protecting field insulators are produced by means of the Low-Pressure Chemical Vapor Deposition (LPCVD) process.

Subsequently, ohmic source and gate metal contacts must be fabricated. This is achieved by opening windows in oxide film, usually during the dry RIE process, and then by PVD deposition of contact metallization in the created windows. Finally, the backside contact metallization to the n+ bulk of the SiC substrate is also deposited and then annealed during the RTP process (~1000°, Ar or Ar/H_2_ atmosphere) so as to thermally form ohmic drain contacts [6]. The manufacturing of high-quality, time-stable ohmic contacts is particularly critical for the operating conditions typical for power devices. We decided to present only a short summary of the recent results in the field of fabrication of both ohmic and Schottky contacts, which are important in SiC technology. The issues of producing metallic contacts in SiC technology are provided with a short introduction to the preparation of the silicon carbide surface, which is essential, or even crucial, in manufacturing the highest quality electrical contacts.

Finally, wire bonds are fabricated while the substrate’s surface is covered with the protective polyimide coating. The high operating frequencies of SiC power devices set strict requirements in terms of admittance parameters related to assembly technology. For this reason, it was decided to include the topic in a separate section, presenting our own experience in the development of assembly technologies for mission-critical and space applications.

## 3. Ion Implantation

Due to the high energy of crystalline bonds in SiC, the formation of regions with various conductivity types differs significantly from silicon technology. Dopant diffusion is practically impossible, so ion implantation must be used to create n- or p-type regions. Moreover, defects created in the course of the implantation process tend to accumulate, and at higher doses, they can even lead to irreversible amorphization. Therefore, implantation in SiC is usually carried out at an elevated temperature (e.g., 500 °C). In addition, dopants in SiC require a higher activation temperature than in the case of silicon doping, even greater than 1800–1900 °C. Post-implantation annealing at such high temperatures requires additional measures to prevent surface degradation. The design and technology of SiC devices must also consider the phenomenon of the incomplete ionization of dopants, associated with high ionization energy (over 200 meV for aluminum acceptors). As in the case of silicon technology, ion implantation is also used in SiC for the fabrication of junction termination areas, but due to the negligible diffusion of dopants, other design efforts or special care must be taken to adequately increase the breakdown voltage. These issues, with reference to the current state of the art, will be briefly discussed in this section. In addition, our experimental results on the production of the junction termination extension area in p-i-n SiC diodes in the 1.7 kV voltage class will be presented.

### 3.1. Dopant Activation

After the implantation process, many dopant atoms are placed in interstitial positions, but to become donors or acceptors, they must substitute atoms in the crystal lattice. If a concentration of implanted dopants (let us consider acceptors here) is denoted as $N_{A}^{*}$, and electrically active acceptors after activation as $N_{A}$, then we can define the activation ratio as $A=N_{A}/N_{A}^{*}$. Activation may occur because of the post-implantation high-temperature annealing (PIA). In general, the activation of donors is less problematic. Depending on the implantation dose, achieving a similar acceptor activation degree requires PIA at temperatures higher by 100–300 °C than those necessary for donor activation at the same concentration levels [7]. For a dopant concentration of the order of $10^{18}{\;\mathrm{cm}}^{-3}$, an observable activation (A=10–20%) takes place at 1400 °C for N and at 1550 °C for Al [8]. Some researchers explain this by the fact that substituting a Si atom with an Al atom (which is necessary to form an acceptor center) requires more energy than substituting a C atom with a N atom (which is necessary to create a donor center), as formation energy values for Si and C vacancies are 8 eV and 5 eV, respectively [9,10]). On the other hand, however, this does not explain why P activates more easily than Al, although it also substitutes Si atoms. This issue is thoroughly discussed in [11]. Many studies indicate that in order to obtain a considerable activation ratio at high dopant concentrations (e.g., $>10^{19}\;{\mathrm{cm}}^{-3}$) for both donors and acceptors, high temperatures have to be applied, such as 1800 °C and higher. It is comprehensively discussed in [12], where a model is proposed, which allows for the prediction of the dopant activation ratio depending on its concentration as well as on the temperature and duration of the PIA. It should be pointed out, however, that this is based on results obtained by the authors, who used different methods for the determination of A, and therefore can be less accurate. PIA is usually carried out in a horizontal furnace, but other approaches also exist, like heating with lamps [13], and local heating with high power density, e.g., PlasmaJet [14,15], or with a pulsed laser [16]. In [17], however, it is pointed out that rapid thermal annealing techniques are more time-consuming and hard to scale.

**Figure 4:**
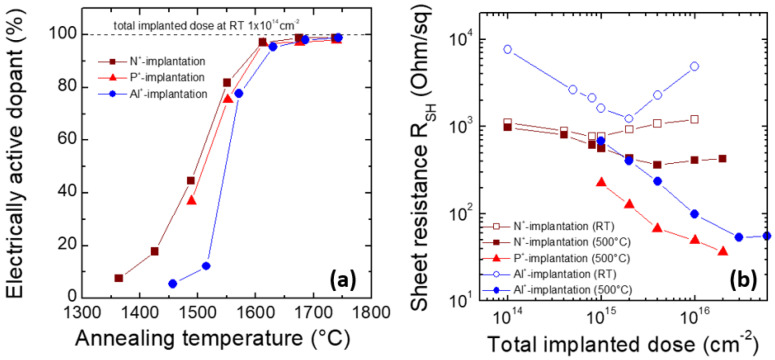
Activation ratio vs. temperature dependence (**a**) and resistivity of the region obtained during implantation at RT and at the elevated temperature (**b**). Reproduced with permission from [7].

Since the activation process is not immediate, an interesting issue is its time characteristics. Discussion of this subject can be found in [8,13,18,19,20,21]. The activation ratio is not only influenced by the process temperature alone, but also by the rate of sample heating and, in particular, cooling [14,15]. It was pointed out that although a higher heating/cooling rate lowers the resistivity of doped regions, it also increases their surface roughness [22]. However, quite opposite conclusions are presented in [13]. The theoretical time characteristic of the dopant activation process contains the model presented in [12]. The standard duration of the process carried out in a horizontal furnace at temperatures of 1600–1800 °C is about 30 min [23,24] and decreases with the temperature rise at 5 min to 1950 °C [25]. The activation ratio dependence on the PIA temperature is shown in Figure 4a.

PIA affects not only the activation of the dopants but also the regeneration of the crystal structure. During the process, parts of the defects can be fixed, resulting in a lower compensating center and higher mobility. Especially for higher doses, crystal degradation could be so strong that regeneration during PIA would be impossible. For this reason, implantation in high temperatures is often needed. The differences between resistivity after implantation at room and elevated temperatures are shown in Figure 4b [7,26].

### 3.2. Surface Consideration During PIA

The main technological problem related to the PIA is the roughness increase resulting from the evaporation of Si atoms. Initial attempts to conventionally anneal implanted samples in the argon or hydrogen atmosphere at normal or lowered pressures led to strong surface degradation due to the defect displacement and Si atom evaporation [27,28]. Various measures were taken to prevent this phenomenon. One of them is covering the SiC substrate with another one while annealing in order to stop the escape of Si atoms to the atmosphere, which results in substantial improvement of the surface quality after the process [27,29]. A modification of this idea is the annealing in the SiC crucible [17]. Another concept is using an AlN cap, which allows us to carry out annealing at temperatures up to 1600 °C (unfortunately, at higher temperatures, the AlN layer starts to form pores) [30]. Also, a BN/AlN double layer was used for this purpose since it enables annealing at 1700 °C (theoretical thermal resistance of BN is 2000 °C) without step bunching [31]. A much easier way of ensuring Si overpressure during the post-implantation annealing is performing this process in the silane atmosphere, which should result in the suppression of Si evaporation. This has already been experimentally confirmed, but some data suggest that the efficiency of this process is due to lower temperatures exceeding 1600 °C [32,33]. The most popular solution is to use a carbon cap during annealing, which shows high thermal stability and is easy to obtain by means of photoresist spinning followed by pyrolysis and may be removed in an oxygen plasma [8,23,26,34].

### 3.3. Incomplete Acceptor Dopant Ionization

The concentration of carriers in the p-type implanted region does not only depend on the implantation dose. It depends primarily on the density of compensation centers already present or formed in the course of the process, as well as on the ionization degree, which remains equal to the ratio of ionized acceptor to all acceptor dopants: $I=\frac{N_{A}^{+}}{N_{A}}$. For acceptors in SiC with an ionization energy above 200 meV, a significant part of the active dopants remains not ionized at room temperature. Designing a region with a given hole concentration should take into account the correction for the ionization ratio. However, it is hard to anticipate the hole concentration at a given dopant concentration, because the relationship is described by an implicit formula:

$$p+N_{D}=\frac{N_{A}}{1+p/x}$$

where $N_{A}$ and $N_{D}$ are acceptor and compensating donor concentrations, respectively, and x is a function of ionization energy, temperature, and $N_{A}$ [35]. Temperature-dependent Hall measurements usually used to determine p as well as $E_{A}$ values also do not give results directly because one must take into account the variability of the Hall coefficient [36,37], or implement a calculation method that takes into account excited state energy levels [38].

**Figure 5:**
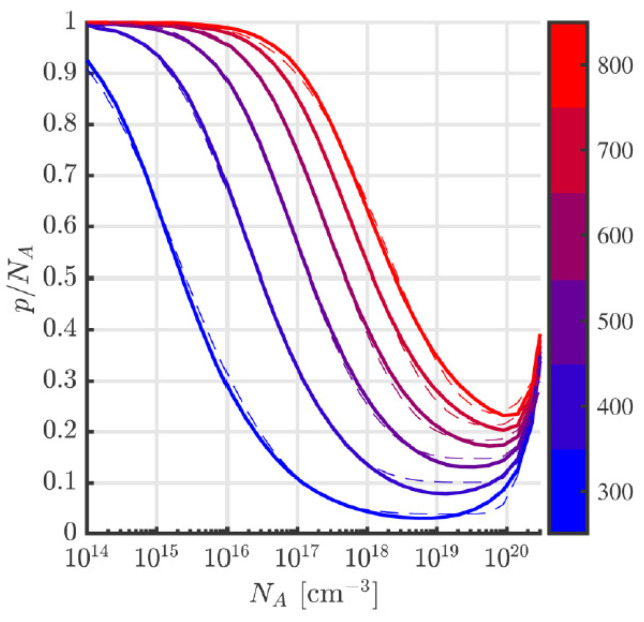
Incomplete ionization ratio calculated using theoretical model at elevated temperatures (solid line) compared to parameterization of empirical data (dashed). Reprinted from [40], with permission from AIP Publishing.

The volume concentration of carriers (in cm^−3^) may be lower by at least a few or even a few hundred cubic centimeters than the number of theoretically electrically active dopants. Therefore, obtaining a region with a high carrier concentration requires high implantation doses, which in turn does not always help, as, at least in the first approximation ionization degree, it drops with an increase in dopant concentration [39]. By considering the electrical field screening of an atom by other free carriers, the so-called band-tailing, it is possible to create a model that anticipates the rise in ionization degree above a certain dopant concentration. For example, a model is presented in [40], according to which the ionization degree at room temperature reaches a minimum (approximately 3%) for dopant concentrations slightly below $10^{19}\;{\mathrm{cm}}^{-3}$ and then increases, reaching concentrations of the order of $10^{20}\;{\mathrm{cm}}^{-3},$ a similar value as for concentrations of the order of $10^{17}\;{\mathrm{cm}}^{-3}$, i.e., around 10%. The results published so far indicate that Darmody’s model can be used and allows for predicting the resistivity and Hall coefficient for Al-doped 4H-SiC layers [41,42,43,44]. The results of Darmody’s model are presented in Figure 5 for a wide Kelvin temperature range. The phenomenon of incomplete ionization does not have a negative impact on the performance of a junction termination (because, in reverse bias, the electric field ionizes all impurities—at least in a steady-state condition), but it does constitute a significant limitation in the possibility of controlling the level of chemically activated impurities by means of Hall effect measurements [36,37,38].

### 3.4. Implantation Process for Junction Termination Extension (JTE)

**Figure 6:**
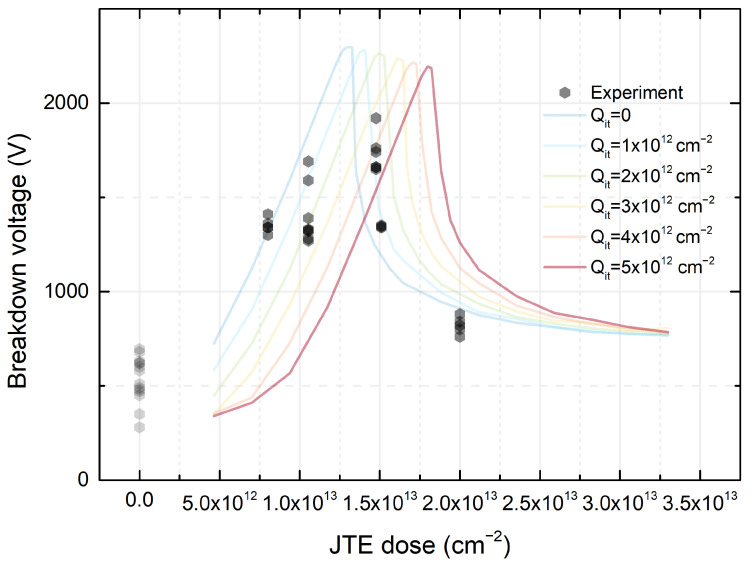
Dependence between Vbr and implantation dose which was used for single-zone JTE fabrication. A dose of zero corresponds to the structures without JTE. Experimental points are shown as individual marks; the curves were obtained by simulation with various effective surface charges.

In our work, a JTE for a 1.7 kV p-i-n SiC diode was fabricated. At the edges of the electrical contacts, a phenomenon of electrical field crowding takes place, which results in electrical breakdown at lower voltages than in the case of an infinite junction. From silicon technology, a well-known method of electric field spreading is a horizontal widening of a p-type region with decreasing dopant concentration. Such a structure is typically obtained via diffusing out an implanted dopant using a carefully prepared mask, the so-called junction termination extension (JTE) [45]. However, for the silicon carbide, this solution is not applicable, but it may be approximated by implanting the region around the junction with a carefully matched dose of acceptors [46]. An even better result may be obtained by dividing the JTE region into two or more zones with the acceptor concentration decreasing outside; in this case, the resulting structures will also be more resistant to fluctuations of the implanted dose as was shown in the previous works [47,48]. From a technological point of view, three-zone JTE can be performed by a double implantation process (doses from inner to outer region are A + B, A, and B, assuming A is dose greater than B), and increasing the number of zones beyond three is uneconomical. Adding implanted rings in the outer region of the JTE zone (space-modulated JTE) requires only the design of an appropriate mask, although the limitation is the achievable critical dimension that can be obtained and the tradeoff between the increased efficiency of JTEs and the area occupied by it. For JTE purposes, the required implantation dose is about $10^{12}\;{\mathrm{cm}}^{-2}$, which means that for a few-hundred-nanometer-deep JTE regions, dopant concentration does not exceed $10^{17}\;{\mathrm{cm}}^{-3}$. Therefore, its ionization should not be difficult, and due to the fact that, for reverse biases, dopants are ionized due to a high electric field, their partial ionization is not a problem. Nevertheless, fabrication of the JTE demands precise control of the implantation dose to work properly [47]. In our work, diodes were fabricated with 60 μm JTEs with various implantation ${\mathrm{Al}}^{+}$ doses between $7.5\;\times \;10^{12}\;{\mathrm{cm}}^{-2}$ and $2.0\;\times \;10^{13}\;{\mathrm{cm}}^{-2}$. For comparison, diodes without the JTE were fabricated as well. In Figure 6, the dependence between breakdown voltage and JTE dose is shown. The lines show shapes of the breakdown voltage dependences for this structure with various effective charges on the dielectric–semiconductor interface obtained by ATLAS/ATHENA simulations. The results reflect the theoretical relationship, taking into account the effective charge on the dielectric interface at the level of about $1\;\times \;10^{12}\;{\mathrm{cm}}^{-2}$, which is consistent with the results of the capacitive-voltage characterization MOS test structures. Although the maximum value is about 10% less than the theoretical one, the structure was, in fact, different from the specification which may explain this discrepancy. The concentration of dopants in the drift layer measured by C-V was $1.5\;\times \;10^{16}\;{\mathrm{cm}}^{-3}$ instead of $7.5\;\times \;10^{15}\;{\mathrm{cm}}^{-3}$ as in the specification. A single-zone JTE is impractical as the dose should be precisely controlled depending on the material, dimensions, dielectric charge, etc. Therefore, design modifications (additional zones and rings at the edge of the junction with the appropriate level of active dopants according to our previously published simulation results [47]) were made to broaden the process window. Also, in our work, diodes with three-zone JTE assisted by three guard rings were fabricated. In the case of these structures, the target breakdown voltage (more than 1.7 kV) was achieved for both $1.5\;\times \;10^{13}\;{\mathrm{cm}}^{-2}$ and $2.0\;\times \;10^{13}\;{\mathrm{cm}}^{-2}$ JTE doses. For lower JTE doses, breakdown voltages on devices with single-zone JTEs and more complicated JTEs were similar, which is in good agreement with simulation results [47]. The technology of the diode fabrication was as follows: sample preparation, mesa fabrication, first JTE implantation, second JTE implantation, channel stopper implantation, post-implantation annealing, oxide fabrication, opening window in dielectric, anode and cathode ohmic contact formation, polyimide passivation, and metallization. The diode diameters were 100, 400, and 800 μm. The target breakdown voltage (more than 1.7 kV) was achieved with the respective 88%, 65%, and 25% yield values. The largest structures conducted about 20 A current with about a 10 V voltage drop (50 ms pulse and 0.1% pulse duty factor), which corresponds to about $4\;{\mathrm{kAcm}}^{-2}$. A more detailed discussion of ways to increase the breakdown voltage value can be found in [46,49,50]. This technology, as well as other issues related to the fabrication of structures showing higher breakdown voltages (with or without implantation), are presented in [50,51,52,53,54,55].

### 3.5. Conclusions

Precise control of the concentration of acceptors, let alone holes in SiC technology, is still not a well-understood issue. Although technologies have been developed to produce low-resistive p-regions, understanding the processes involved in the formation of such layers and the characterization technology still requires further research. The inability to precisely control the concentration of acceptors also limits the use of a simple structure of the JTE. In our own work, high-current-density 1.7 kV pin SiC diodes were fabricated with diameters up to 800 μm with a JTE region obtained by the implantation of Al ions. The fabricated structures were characterized in a forward direction by the pulse method up to 200 W.

## 4. SiC Dry Etching

One of the most crucial technology operations is the formation of a specific profile in the substrate to optimize the breakdown voltage of power devices. The process should be selective with respect to the masking material to provide an appropriate etching rate, anisotropy, and minimum damage level of the processed surface. The presented SiC etching technology is a result of our process optimization efforts aimed at obtaining MESA structures used subsequently for the PIN diode fabrication. MESA is a spatial structure of a specific shape, height, and sidewall angle. During experiments, a lot of attention was paid to the optimization of the MESA sidewall angle. Two methods of dry etching were investigated. The first one was reactive ion etching (RIE) which offers high anisotropy and good control of process parameters, which in turn results in its high reproducibility [56,57,58,59,60,61]. The second technique was inductively coupled plasma reactive ion etching (ICP-RIE) [62,63,64,65]. In general, ICP-RIE processes allow us to obtain higher ionization degrees of plasma (i.e., plasma density) than the standard RIE processes, which is advantageous.

### 4.1. RIE

**Figure 7:**
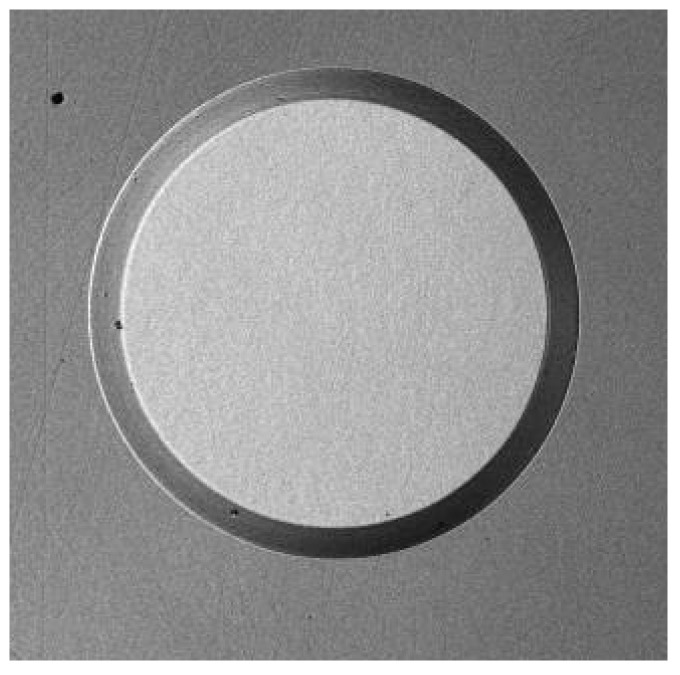
The 4H-SiC substrate with a SiO_2_ mask.

Plasma-enhanced chemical-vapor-deposited (PECVD) SiO_2_ was used as a mask during RIE [59]. The pattern transfer into a “hard mask” was obtained by wet etching in hydrofluoric (HF) acid. MIR 701 photoresist with a thickness of 1.5 μm was used for the silicon oxide patterns. Figure 7 presents the 4H-SiC substrate with a SiO_2_ mask. These structures were then subjected to the RIE. The processes were carried out using a PlasmaLab 80+ reactor from Oxford Instruments (Abingdon, UK) with a 13.56 MHz RF power supply with power adjustable in the range of 50–300 W and operating at a pressure above 30 mTorr. The SF_6_ and O_2_ gas mixture was chosen for plasma creation, as it resulted in a faster etching rate and better surface quality (lower roughness) of SiC than the CF_4_ and O_2_ gas mixture [57,66]. Subsequent investigations allowed us to determine the influence of the RF power, gas pressure, and flowrate on the SiC etching rate. Table 1 shows selected results for etch rates for SiC and the hard mask (SiO_2_) as a function of etch process parameters.

**Figure 8:**
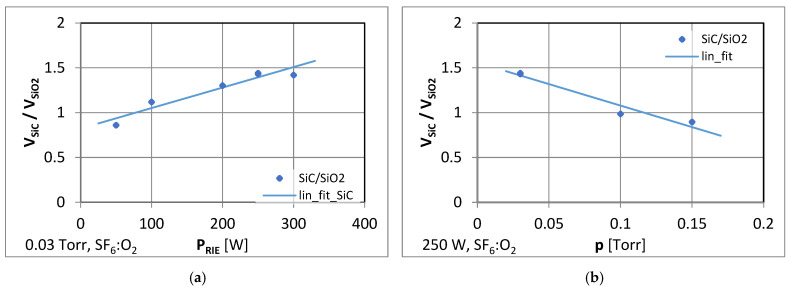
Selectivity of SiC etching over SiO_2_ in SF_6_ + O_2_ plasma as a function of (**a**) the plasma P_RIE_ power at p = 30 mTorr and (**b**) the plasma pressure at P_RIE_ = 250 W.

The ratio of SiC substrate removal (Vs_iC_) to SiO_2_ mask removal (V_SiO₂_) was established. At the constant pressure of 30 mTorr for the SF_6_ and O_2_ plasma gas mixture (flowrates of 20 sccm and 30 sccm, respectively), it was determined that at the RF power ranging from 50 W to 300 W, the ratio of V_SiC_/V_SiO₂_ varied between 0.9 and 1.43. The dependences of SiC and SiO_2_ etching rates on the RF power variation are shown in Figure 8a. The V_SiC_/V_SiO₂_ ratio close to 1 was obtained at the power of 70 W. In Figure 8b, the influence of plasma gas mixture pressures on SiC and SiO_2_ etching rates are presented. The ratio of V_SiC_/V_SiO₂_ close to 1 was obtained at the pressure of 120 mTorr and an RF power of 250 W.

**Figure 9:**
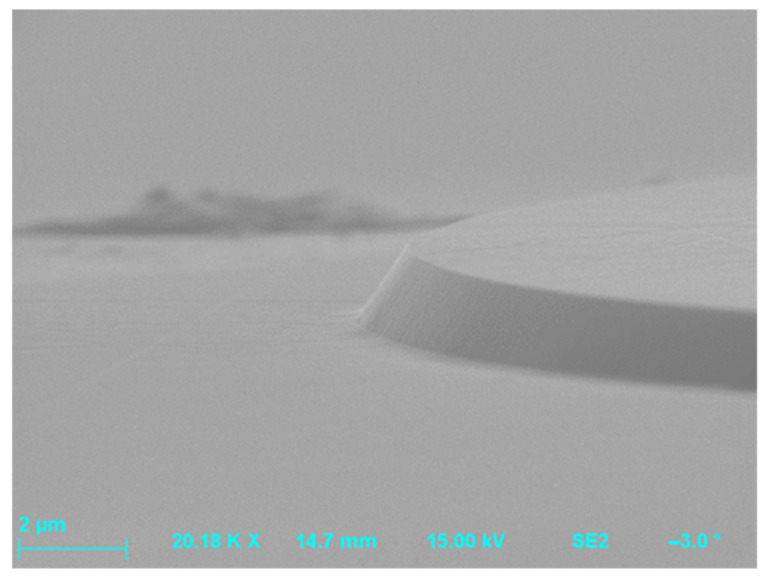
SEM image of the MESA structure obtained in the course of the RIE of 4H-SiC (at P_RIE_—300 W; O_2_ flowrate—30 sccm; SF_6_ flowrate—20 sccm; and p—30 mTorr). The height of this MESA structure was around 1.35 µm.

Precise control of etching selectivity enabled us to obtain the appropriate sidewall angle of the MESA structure. Analysis of experimental results led to the optimization of the RIE process parameters and, as a consequence, to obtaining 4H-SiC MESA structures with no trenching effect, a sidewall angle of 63°, and a surface roughness (R_RMS_) of 1.56 nm (Figure 9).

These results show that the RIE method is usable in the fabrication of MESA-type structures. It can be an alternative to the ICP-RIE method which is described in Section 4.2. The etched SiC bevel structures with a wall inclination angle of 40–80° may be used, for instance, in the fabrication of the epitaxial layer of AlGaN/GaN/SiC diodes and transistors. An angle of ~60° is preferable, as it is less prone to peripheral breakdown [67].

### 4.2. ICP-RIE

Another method other than RIE that is better for deeper and high-aspect-ratio etching of SiC is inductively coupled plasma reactive ion etching (ICP-RIE). In the ICP-RIE system, the plasma is generated with an RF-powered magnetic field, and another RF generator is used to direct the reactants toward the substrate using an electric field. The standard ICP-RIE PlasmaPro100 reactor (Oxford Instruments Ltd.), equipped with two 13.56 MHz RF power supplies, was used. The power of RIE and ICP generators was changed independently, as well as the reaction gas flows and the pressure inside the chamber. ICP-RIE allows us to perform a deep etching, but it also can produce some undesirable effects:

(1) Trenching and micro-trenching, which are related to the formation of irregularities at the bottom of the SiC-etched trenches or on the sidewalls of MESA structures;
(2) “Undercutting” of sidewalls of produced structures in the processes with a dominant chemical etching mechanism;
(3) Formation of the micro-trench tips at the bottom corner of the sidewalls, which can cause local growths of electric field and degrade the breakdown characteristics of power devices;
(4) Micro-masking, which is related to the redeposition process of non-volatile chemical compounds on etched surfaces (caused by impurities or imperfections, e.g., native oxides, dusts, or scratches of the surface as well as the erosion of various metallic masks used in the process).

**Figure 10:**
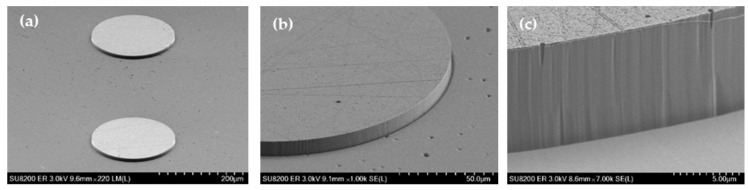
SEM images of MESA structures obtained after the ICP-RIE of SiC with the Cr mask in the SF_6_ plasma (no micro-trenching effect on the MESA sidewall is visible) performed with an magnification of (**a**) 220× (**b**) 1000× (**c**) 7000×. The etching process parameters were SF_6_ flowrate = 100 sccm, P_RIE_ = 50 W, P_ICP_ = 2500 W, p = 7 mTorr, and t = ~18.5 min (a time of the total Cr mask etching).

A detailed discussion of the phenomena listed above can be found in the recently published review paper on the ICP-RIE of SiC [68]. The surface quality of etched structures is a critical factor in the fabrication of SiC devices. Hence, it is important to determine the appropriate parameters of the etching process so as to eliminate the micro-masking effect and/or formation of residual structures. Examples presented in the literature show that the formation of undesirable structures can be limited, for example, by increasing the pumping rate of the chamber, increasing the temperature of the etched sample, or gradually changing the etching conditions simultaneously with the etching depth being obtained [69,70]. The kind of mask used in the etching process is a key parameter in the development of SiC structures with desired properties. The use of metallic masks in the ICP-RIE process, such as a Cr or Ni mask, allows for etching deep patterns and obtaining structures with a large wall inclination angle (~90°), which can be used, for example, in the production of diodes, transistors, and switches. Figure 10 shows an example of ~8 μm height MESA structures obtained after SiC etching with the use of a Cr mask, for which smooth, vertical walls were obtained. In this case, the duration of the etching process was defined as the time of the total etching of the Cr mask. This time was determined by the endpoint detection procedure [71] implemented in the software of the reactor (the endpoint panel of the PC4500 software), so in Figure 10, after the etching process, the SiC surface morphology with high roughness is visible. To prevent such severe roughness, the etching should be completed before the metal mask is removed. The Cr mask can be removed with a dedicated Cr etching solution or hydrochloric acid (HCl), depending on the application. The use of non-metallic masks, e.g., photoresist or SiO_2_ masks, can be useful in “shallow” etching processes, in which patterns with a small wall inclination can be obtained.

**Figure 11:**
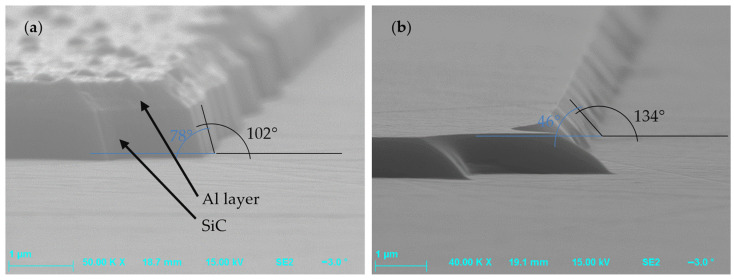
Images of the SiC surface after ICP-RIE processes with two different types of masks: (**a**) the aluminum mask and (**b**) the AZ 4562 photoresist.

Etching processes that showed differences in the etch patterns, resulting from the use of non-metallic (photoresist) or metallic masks, are shown in Table 2. The first mask type was the classical photoresist AZ 4562 which allows us to obtain masking layers thicker than 5 µm, which in turn enables us to achieve an etching depth of 1.3 µm. The hard mask effects were studied using an aluminum (Al) 0.5 µm thick layer produced by magnetron sputtering. Figure 11 presents SiC surfaces obtained for both Al and photoresist AZ 4562 masks after etching processes performed at the same experimental conditions: SF_6_ flowrate = 20 sccm, O_2_ flowrate = 2 sccm, P_RIE_ = 100 W, P_ICP_ = 900 W, p = 7 mTorr, and t = 10 min. In this figure, it can be seen that the Al mask allowed us to obtain steep sidewalls of the etched structures, with the sidewall angle larger than 75°. However, there was a problem when using this mask, i.e., the micro-masking effect appeared. During the etching process, the etched material from the mask, which was not evacuated by pumping, was deposited on the exposed surface. Thus, the Al mask proved unsuitable for PiN diode technology. On the other hand, the use of the AZ 4562 photoresist mask enabled us to obtain good quality MESA structures, with a sidewall angle of ~46°.

Investigations that allowed us to determine the influence of the ICP etching process parameters on the MESA sidewalls’ quality and angle were also performed. It is well known that the main ICP-RIE process parameters are pressure in the reaction chamber, RIE power, ICP power, etching time, and flowrate of plasma-forming gases. From our many etching processes performed, it turned out that both *P*_RIE_ and *P*_ICP_ powers, as well as the working gas composition, are crucial from the viewpoint of controlling the sidewall angle of the MESA structure.

**Figure 12:**
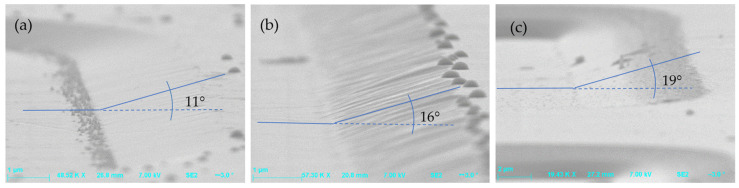
SEM images of SiC MESA structures after the ICP-RIE of SiC with photoresist AZ 4562 mask, with various RIE powers: (**a**) 100 W, (**b**) 200 W, and (**c**) 300 W. The pressure in the reactor was 7 mTorr and the ICP power was 800 W. The gas flowrates: SF_6_—18 sccm; O_2_—9 sccm.

**Figure 13:**
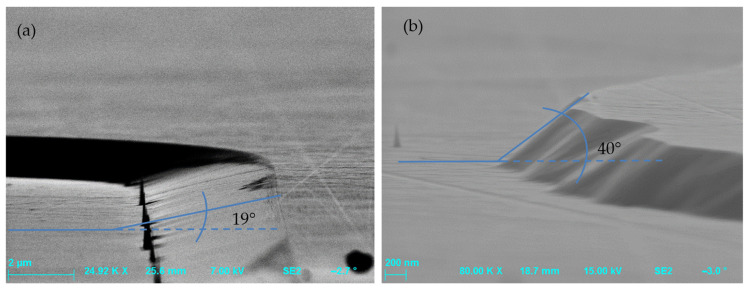
SEM images of SiC structures after the ICP-RIE of SiC with photoresist AZ 4562 mask, with various ICP powers: (**a**) 800 W and (**b**) 1300 W. The pressure in the reactor was 7 mTorr and the RIE power was 100 W. The gas flowrates: O_2_—9 sccm; SF_6_—18 sccm.

The influence of the RIE power on the inclination angle of MESA structures’ sidewalls (after etching of SiC with a photoresist AZ 4562 mask) is shown in Figure 12. It can be seen in this figure that increasing the RIE power results in increasing the sidewall angle, which is at the level of a few degrees. On the other hand, Figure 13 shows the influence of the ICP power on MESAs’ sidewall angle. One can observe in this figure that an increase in ICP power is accompanied by an increase in the sidewall angle of MESA structures, and for an ICP power of 1300 W, this angle is around 40°.

**Figure 14:**
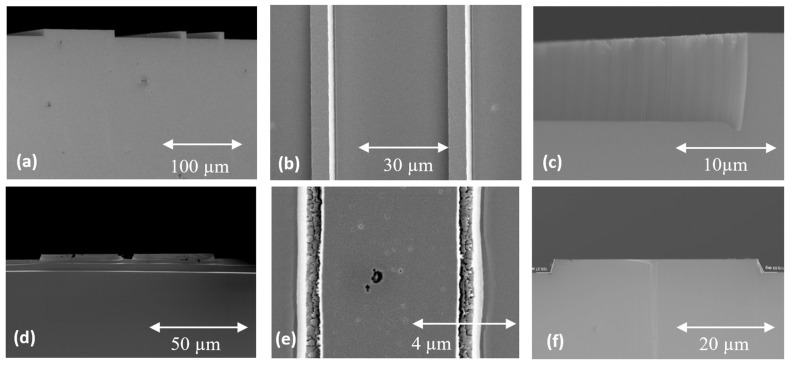
SiC trenches obtained after ICP-RIE in pure SF_6_ plasma (photos at the top; P_ICP_ = 2250 W, P_RIE_ = 50 W, p = 7 mTorr, gas flowrate SF_6_ = 100 sccm) and in SF_6_ + 50% O_2_ (photos at the bottom; P_ICP_ = 2250 W, P_RIE_ = 50 W, p = 7 mTorr, gas flowrates: SF_6_ = 50 sccm and O_2_ = 50 sccm). (**a**,**d**) View of the structure profile; (**b**,**e**) SiC surface morphology; (**c**,**f**) view of sidewalls with the inclination angles of ~90° and ~63°, respectively.

As mentioned above, the working gas composition is also a crucial process parameter from the viewpoint of controlling the sidewall angle and the surface morphology of the MESA structure. In our recently published review paper on the ICP-RIE of SiC [68], we presented detailed experimental results of the etching of SiC with the Cr mask including, among others, the influence of the gas atmosphere (which was SF_6_ + O_2_ with various oxygen contents) on the SiC etching rate, the etching rate of the Cr mask, and the selectivity of the etching process. In this paper, a continuation of our previous investigations is presented; namely, Figure 14 shows the SEM photos of SiC structures obtained after the ICP-RIE with various oxygen contents in the SF_6_ + O_2_ plasma: 0% O_2_ and 50% O_2_, respectively. From these figures, it can be concluded that the oxygen admixture influences the SiC surface morphology and also the sidewall inclination angle. Increasing the oxygen content in SF_6_ + O_2_ plasma introduces the sidewall micro-trenching effect (Figure 14c) and surface roughness (Figure 14e) and also reduces the sidewall angle from ~90° for 0% O_2_ (Figure 14c) to ~80° for 5% O_2_ (not shown here) and then down to ~63° for 50% O_2_ (Figure 14f).

### 4.3. Conclusions

In conclusion of this section, we can state that the ICP-RIE method, which combines high finishing accuracy and reproducibility, proves to be excellent for etching deep patterns in the form of vertical, smooth walls in SiC. There are a number of aspects that should be taken into account when designing processes aimed at obtaining spatial SiC structures with the desired properties. Experimental investigations presented above show that it is important to select appropriate conditions for the etching process and that the key parameters are RIE power, ICP power, the chemical composition of the plasma, and the type of mask used in the etching process. Properly developed SiC etching technology allows for elimination of the undesirable effects, such as trenching, micro-trenching, or micro-masking, and for obtaining etched SiC structures of a high quality with smooth surfaces and with the desired sidewall angle.

## 5. Thermal Oxidation

As a material intended for power electronics, silicon carbide has many advantageous technological and physical properties (wide bandgap, high critical electric field, and high thermal conductivity) [39,72]. As far as technological issues are concerned, SiC, in contrast to other wide bandgap materials, possesses attractive features, including its ability to form a dielectric in the process of thermal oxidation. The SiO_2_ layer produced in this process has excellent insulating properties in the SiC/SiO_2_ material system [39]. However, in contrast with the silicon-based thermal oxides, the oxide produced by SiC oxidation exhibits significant issues with the quality of SiO_2_ dielectric layers when compared to a similar layer obtained in silicon technology [73,74,75]. The main difference is the high amount of charge trapped near the electrically active SiC/SiO_2_ interface that can be exchanged with the semiconductor. This results in two main application obstacles: The first one is the influence of such a dielectric when used as gate oxide on the conductive channel of the MOSFET transistor—the key-switching device in power electronics. A large amount of charge trapped in the dielectric subsurface layer leads to high carrier scattering in the transistor’s channel, causing a decrease in the effective channel mobility and increasing channel resistance in a fully turned-on MOSFET [76,77]. The second problem is the high density of traps in oxides used to passivate the power devices, particularly in the case of junction termination that is required for the operation of such devices at higher blocking voltages. Charges accumulated in the passivating layer may modulate the distribution of the electric field in the structures intended to protect the diode against breakdown (e.g., in JTE structures) [78,79]. As a result, they may not fulfill their role and thus limit the applicability of such devices.

This section provides a comprehensive description of the physical causes of these problems and known technological solutions that can be applied to improve the quality of oxides manufactured in SiC oxidation. The state of the art and the established technological solutions for those problems are also presented and discussed in detail.

### 5.1. Important Interface Defects

Due to its two-component structure, the progress of the reaction of the oxidation in the case of SiC is complicated and can run in many ways. The complex nature of the two-compound material allows for several oxidation reactions to occur at the interface. The most important reactions for the production of bulk oxide are those with the lowest activation energy. The literature [80,81,82] considers the reaction of molecular oxygen (O_2_) in the state of the lowest possible energy (triplet oxygen) which has crossed the diffusion barrier. In this case, Knaup [82] has found that there are two preferable reactions leading to the production of bulk oxide. The reaction is always performed in three steps and requires two oxygen molecules. The first step is the oxygen molecule being incorporated into the interface by the injection of the carbon atom into an interstitial position (V_C_O_2_) and forming a carbon interstitial (C-C_i_). Another slightly less favorable reaction is the formation of a Si-O-C bridge and the release of the oxygen atom as an interstitial 2(O_i_^if^). At this stage, there is no carbon emission from the vicinity of the interface yet. The second step occurs when another oxidation molecule reaches the interface. Then, either the above-described reactions take place, or such a molecule reacts with the previously created structures to form a CO molecule. This molecule then diffuses towards the oxide surface and leaves the defect complex (O_i_^if^ + V_C_O_2_) behind. Finally, the third molecule produces stoichiometrically complete oxide by emitting another CO molecule.

Although those reactions are energetically favorable, a number of other paths exist that can lead to the creation of stable defects at the interface. First-principle simulations suggest that these are various forms of stable carbon defects, mostly carbon dimers (C_i_)_2_ or other larger carbon aggregates, carbon interstitials, carbon dangling bonds, carbon C-C bonds, or carbon vacancies. Recently, Akiyama et al. [80] presented a similar oxidation process of oxygen incorporation via a metastable state, which leads to the formation of a double silicon bond Si_2_-CO, and the subsequent release of the CO molecule via structural relaxation. This work also pointed out that the most common defect types at the SiC/SiO_2_ interface are carbon-related defects, preferably in the form of carbon interstitials, carbon dangling bonds, carbon C-C bonds, or carbon vacancies. It has been shown that such defects do not diffuse deep into SiO_2_, being mobile only in the area of the SiC/SiO_2_ transition region [83]. A defect of type (C_i_)_2_ is a carbon defect consisting of two interstitial carbon atoms bound together (carbon dimer). This defect is of great importance, as unlike the (C-C_i_)_C_ defect, it is stable [82,84,85,86,87,88]. For this reason, it has been indicated in many studies as a dominant source of trap states affecting the operation of the MOSFET structures.

Although the defects described above form and reside at the SiC/SiO_2_ interface, many of them, especially stable defects like carbon dimers, may remain in the oxide volume as the oxidation proceeds. This mechanism is attributed to the fact that apart from the quickly reacting interface traps in SiC, a significant number of traps with large time constants are already present in the oxide volume. These defects can still exchange charges with the semiconductor via tunneling. These are the so-called near-interface traps (NITs).

Since the vicinity of the defect has a significant impact on the energy position of the trap associated with a given effect, and due to the amorphous structure of the oxide formed, the interaction of the defects described above with the semiconductor significantly varies depending on how close the defect is to the semiconductor/oxide interface.

### 5.2. Oxide Quality and Electrically Active Defects

The most important problem associated with thermal oxides is the high trap density (D_it_). In this section, we will summarize the origin of the observed trap energy profiles in the bandgap of 4H-SiC (0001) since these types of substrates are most commonly used today.

**Figure 15:**
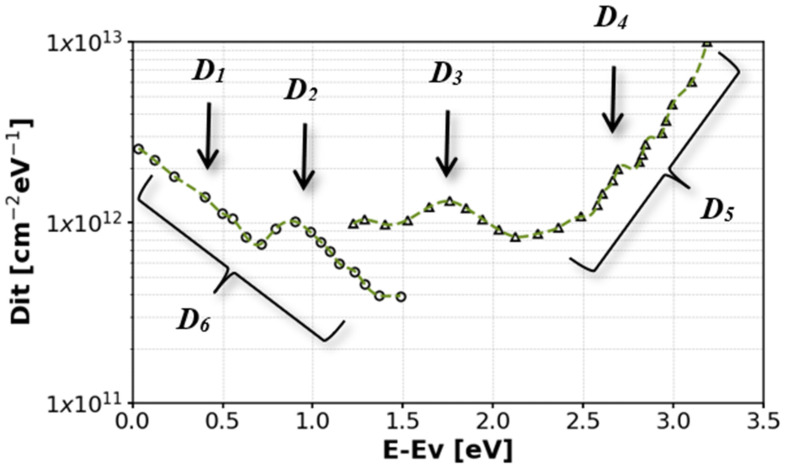
Typical energy distribution of trap state densities across the 4H-SiC bandgap for samples produced in dry oxidation.

The energy distribution of the trap states in the forbidden gap is continuous and U-shaped. The energy distribution in dry thermal oxides for hexagonal polytypes is shown in Figure 15 [73]. One can distinguish several maxima in the trap state density characteristics. The first one (referred to hereinafter as D_1_) is located near the edge of the valence band at a distance of approximately E_v_ + 0.5 eV (see Figure 15) [73,84].

The second is a wide maximum located at an energy of approximately E_v_ + 1 eV (hereinafter referred to as D_2_). Another distinguishable trap peak is located near the center of the SiC bandgap, denoted by D_3_ [73,84]. Another distinct maximum (D_4_) [89,90,91] is highly dependent on the oxidation process conditions. It is generally not present in optimized oxidation technologies such as those described below.

From a practical point of view, the most critical are the blurry, continuous trap state spectra located near the edges of the valence and the conduction bands (D_5_ and D_6_ in Figure 15) because they consist of the highest trap state density and thus, they have the largest impact on the overall trapped charge in the interface. Their location near the edge of bands strongly affects the parameters of the MOS devices made in SiC. D_5_ states are the most significant, as traps in this energy range become acceptors. As can be seen in Figure 15, the natural dry oxide formed during the oxidation process results in trap densities close to 10^13^ eV^−1^cm^−2^ near the edges of the bandgap. As mentioned earlier, this value is high and can cause problems in the transistor or diode operation.

### 5.3. Physical Origins of the Trapped Interface Charges

**Figure 16:**
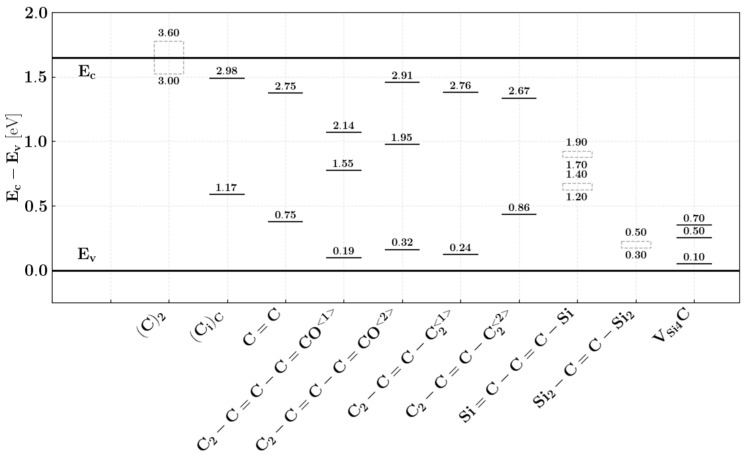
Identified defects in the 4H-SiC–SiO_2_ interface contributing to the active trap profile: <1> without stacking fault and <2> with stacking fault. Based on [88,92,94].

Carbon, the element naturally present in the SiC/SiO_2_ system, is the dominant source of the observed trap states. It was shown that these carbon defects can be attributed to the D_5_ and D_6_ density of state distribution regions. The most troublesome traps—with a blurry energy distribution near the conduction band (D_5_) and valence band (D_6_) edges—have been connected to a variety of carbon-related defects listed in Figure 16. The largest contribution to this high interface trap density region has been attributed to the double-bonded C=C defects [88] since the energy distribution can be explained by the changes in the dihedral angle of such structures at the interface. The lower part of the D_it_ profile near the valence band (D_6_) has been attributed to double carbon in the antiside (C_2_)_Si_ [88,92,93].

It is worth mentioning that the dominant electrically active defects close to the mid-gap are interstitial carbon atoms in oxide defects [88] in the form of type (C_i_)_C_ split interstitial defects that can be attributed to the (D_3_) center. However, the activation energies of these states depend on their neighborhood and thus this defect can also contribute to lower parts of the D_5_ and D_6_ slopes [85].

The lower half of the bandgap, the silicon vacancy near the semiconductor surface surrounded by four carbon atoms (V_Si4_C), can play a significant role in the creation of the D_6_ D_it_ region [94].

### 5.4. Improving Oxide Quality

There are two main approaches to improving oxide quality in terms of trap density. The first one is based on using different add-ons in the oxidation mixture; the most commonly mentioned are nitrogen [95,96,97], phosphorus (P) [98,99,100,101], boron (B) [102], sodium (Na) [103], and barium (Ba) [104] compounds. Only nitrogen in the form of NO or N_2_O is widespread and considered a standard [105]. Therefore, it is highly desirable to find out how these particular processes can affect the aforementioned defect-creation mechanisms. Other add-ons, although discussed in the literature, have a fundamental drawback, as the introduction of P, Na, and Ba have some influence on oxide reliability. Due to the diffusive nature of the entire process, oxide volume is rich in such elements. This can cause significant problems with the threshold voltage stability (in the case of the gate oxides), generation of NIT-type oxide traps, or even degradation of the insulating properties of the oxide layer. Several efforts have been made towards reducing these effects by carrying out technology in such a way so as to lower the amount of the above-discussed elements in the oxide volume [99].

**Figure 17:**
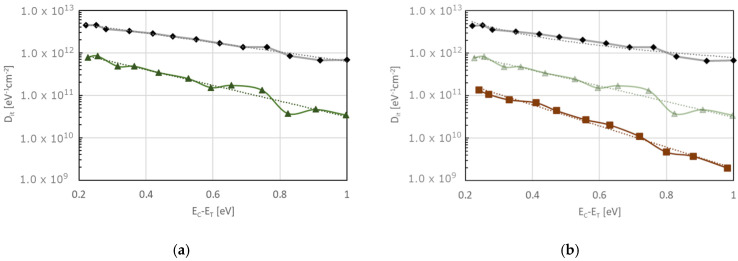
Trap density profiles calculated by the Hi-Lo method (100 kHz) near the conduction band edge of 4H-SiC (0001). (**a**) Black diamonds—simple dry oxidation at 1175 °C; green triangles—a simple dry oxidation followed by the 1000 °C NO annealing step. (**b**) Black diamonds—simple dry oxidation at 1175 °C; brown squares—a simple dry oxidation followed by the POCl_3_ annealing and subsequent NO annealing step at 1000 °C; green triangles—a simple dry oxidation followed by the 1000 °C NO annealing step for comparison. Dashed lines—a fitted U-shaped profile (U6) for each sample. Based on [106].

Typically, nitrogen is introduced into the oxidation process as an add-on to the oxidation mixture or in a separate annealing step using NO gas. Figure 17a shows our original results comparing a trap density profile of dry oxidized samples with and without subsequent NO annealing at 1000 °C using the Hi-Lo method [106].

Nitrogen-oxide annealing is effective in reducing the overall trap density by about one order of magnitude. However, the reduction is greater for traps with energy levels deeper in the bandgap. Nitrogen incorporation has been found to shift the trap energy associated with a given carbon defect outside the bandgap, thus effectively reducing the trap density inside. It was theoretically shown using DFT simulations with respect to carbon-type defects in the form of interstitial carbon atoms (C-C_i_)_C_ [84,94,107]. It has been shown that nitrogen incorporation into the SiO_2_/SiC interface can transform some of the stable carbon dimers into less stable structures, improving subsequent oxidation of those defects and associated traps [108]. It was shown that annealing in NO is particularly effective in removing some forms of double-bonded carbon (i.e., double-bonded interstitial positions [94]) but not all structures equally. As can be seen in Figure 17, this corresponds to the energy location being slightly deeper in the bandgap where the observed D_it_ reduction is greater. Recent studies suggest that some types of silicon defects in the form of Si-Si bonds can be removed by nitridation [109]—mostly the Si dangling bonds and silicon vacancy V_Si4_C [94]; however, those defects play a dominant role close to the valence band edge. It is worth mentioning that the passivation mechanism of a stable carbon dimer defect requires several NO molecules and forms the intermediate steps that shift the trap energy deeper into the bandgap but do not eliminate the trap entirely.

To further improve oxide trap density, other technological processes are required. The most often investigated are the annealing-based processes that can be applied during or after oxidation by adding other elements such as phosphorus or boron to the oxidation/annealing mixture. The most frequently investigated approach is to introduce phosphorus in, for example, POCl_3_ annealing which is compatible with the existing silicon technology. The results of such an approach can be seen in Figure 17b, where a POCl_3_ annealing step has been performed at 1000 °C with subsequent NO annealing also at 1000 °C. The introduction of phosphorus into the system changes the oxide layer into a phosphosilicate glass. This technological step has two main consequences. Firstly, it reduces the trap density profile, and secondly, it creates problems with mobile phosphorus ions, especially at higher temperatures, resulting in thermal instability of the charge distribution in the oxide. Phosphorus incorporation is capable of a significant reduction in the trap density profile for another order of magnitude. It is worth noticing that this step can efficiently reduce the trap state density close to the conduction band. The influence of phosphorus incorporation is still not fully understood. Two main hypotheses are that (1) phosphorus can participate in the interface and near oxide structural reconstruction, promoting the elimination of some defects (e.g., Si–Si bonds) [98,110,111] and (2) some of the C-related defects are removed by the incorporation of P [110,112,113]. Recent investigations suggest that P can weaken the strength of the carbon dimer bond, making it more prone to oxidation and removal. This process can be enhanced by structural changes in the interface layer. It was shown that the reduction in size of the trap density close to the conduction band is proportional to that of interface saturation with P [114].

Charge distribution instability at higher temperatures is still a major issue for the practicality of phosphorus atmosphere annealing technologies. However, several attempts have been reported to reduce this problem by introducing phosphorus via implantation rather than through diffusion [99].

The second approach considered recently is based on the deposition of a thin layer of pure Si on top of SiC and then the performance of low-temperature oxidation of SiC [115,116]. It is free of the defect problems described earlier because SiC does not effectively oxidize at temperatures below 1000 °C [117]. Therefore, it is possible to oxidize the top Si layer in temperatures as low as 750 °C. Since no SiC is oxidized, there is no carbon-related defect creation during this process. It has been shown that this approach in combination with nitrogen annealing can produce D_it_ values close to the conduction band edge as low as 10^10^ eV^−1^cm^−2^.

### 5.5. Conclusions

Macroscopic and physical mechanisms responsible for SiC oxidation and defect formation have been shown and discussed in this section. The main physical reasons for traps in the SiC/SiO_2_ system are carbon defects and excess carbon that has not been removed from the interface. Therefore, a number of technological steps can be applied to neutralize the electric influence of those centers or to promote the removal of carbon compounds by structural changes in the interface. A number of different approaches have been investigated so far, of which NO annealing is considered the standard in modern SiC technology. Oxide produced in NO ambient, an admixture, or with post-oxidation NO annealing retains all benefits of a dry oxide while providing significant trap density reduction. Phosphorus post-oxidation annealing or admixture has been proven to be effective in trap density reduction to an even greater degree than technologies involving NO. However, initial experiments had a significant drawback of producing phosphosilicate glass in the result, which has been unstable in large electric fields due to phosphorus ion mobility in the oxide. This instability in the charge distribution over the oxide volume strongly affected the threshold voltage of the MOSFET transistor as well as other possible applications of such oxides, such as passivation oxide for diode termination. Therefore, it has been considered impractical. However, as has been discussed here, the mechanisms by which those two main approaches influence defects at the interface are distinct and can be combined to further improve SiC oxide quality. This can be achieved by processing modifications that mitigate phosphorus influence over the oxide such as phosphorus implantation or two-step oxidation of SiC with a low-temperature deposited Si oxidation step, which is, to date, the most promising approach. As the technology matures, the quality of oxides is improved to the point where the dominant role of carbon defects has been properly addressed. Therefore, in the future, different types of defects can become more problematic. These silicon-related defects, mostly originating from the oxidation process and lattice mismatch between SiC and SiO_2_ (dangling bonds), can play a dominant role.

## 6. Electrical Contacts

Metal–semiconductor (M-S) contacts may exhibit ohmic or rectifying operations depending on the metal applied and the carrier concentration in the semiconductor, as well as the surface state and thermal treatment. Both, Schottky Barrier Diodes (SBDs) and ohmic contacts are widely used and undoubtedly important structures for microelectronic technology.

Ohmic contacts, which usually function as electric contacts, play a crucial role in any semiconductor device, as their insufficient operation might deteriorate even the most promising outcome of the device’s active structure. Therefore, developing highly efficient ohmic contacts with the highest possible reliability should be one of the main priorities during the design and fabrication process.

Silicon carbide Schottky Barrier Diodes have continuously attracted much attention due to their advanced development and versatile potential. There are plenty of possible applications of Schottky junction design, such as active power factor correction circuits, uninterrupted power supplies, electric motor drives, PV inverters, etc.

M-S contacts may seem simple and undemanding to manufacture, but there are a few crucial points to consider. In addition to appropriately matched materials, there should be a properly adapted way of surface treatment, and ultimately, the parameters of the metallization process will be adjusted to a specific case. Only by developing all these technological issues simultaneously can the expected results be achieved.

### 6.1. Surface Treatment

Surface cleaning is among the most critical processes in semiconductor technology. Surface impurities built in during its processing may cause a reduction in device stability, reliability, performance, and even production yield; a clean and flat surface is extremely important from the point of view of ohmic contact formation or bonding [118,119]. Over the recent decades, much attention has been paid to the development of effective and efficient methods of elimination or at least reduction in the presence of native oxides, organic contamination, residual photoresist, alkali ions, and metallic species. The most common and conventional remedy used to cope with these problems is wafer immersion in chemical baths, often followed by annealing in order to ensure the removal of remaining impurities. The most frequently used chemical solutions for this purpose include the Piranha solution (H_2_SO_4_+H_2_O+H_2_O_2_) and the procedure developed by the Radio Corporation of America (RCA) in 1965, based on a three-stage cleaning in solutions containing hydrogen peroxide, ammonium water, hydrofluoric acid, and hydrochloric acid as active agents. Although the RCA procedure, well known from silicon technology, still remains the most popular cleaning method, sometimes, it is insufficient or even fails under certain conditions. To achieve the expected quality of the final product, it is occasionally necessary to modify classical methods of semiconductor surface cleaning. Frequently, these modifications include combinations of vapor or plasma processes along with a bath in liquid solutions [120,121,122].

### 6.2. Ohmic Contacts

Despite the numerous superior properties of silicon carbide, its potential capabilities cannot be fully exploited without good ohmic contacts. Ohmic contacts to SiC-based devices should exhibit low resistivity, stable electrical conductivity, high oxidation resistance, and reliability, in particular, at elevated temperatures. Insufficient electrical conductivity of ohmic contacts caused by the degradation of the metal/SiC interface, or relatively high specific interfacial resistance ρ_c_ (>10^−3^ Ωcm^2^), may result in an unacceptably high voltage drop at the contact, and therefore deterioration or even the prevention of the operation of devices [123,124]. Although these issues have been investigated for the last two decades, which resulted in some significant achievements, the way of forming acceptable structures in a reproducible way is still being sought, particularly as far as contacts to p-type semiconductors are concerned.

Currently known and commonly applied methods of fabricating ohmic contacts to n-type SiC are considered satisfactory from the viewpoint of commercial manufacturing of devices. Ni and Ni-based ohmic contacts are usually used for n-type 4H-SiC. Due to silicidation at high temperatures (typically 900–1100 °C), Ni/SiC contacts demonstrate low specific resistance (less than 1 × 10^−5^ Ωcm^−2^) and can be obtained in a highly reproducible manner [125,126,127,128]. As nickel reacts mainly with silicon at these temperature levels, unreacted carbon diffuses outwards the interface, resulting in the formation of surface defects. In order to ensure the temperature stability of contacts, attempts have been made to replace nickel with other materials, e.g., Ti, Pt, W, and their alloys [129,130].

In theory, fabricating an ideal ohmic contact to p-type semiconductors requires using a metal with a work function higher than the work function of the semiconductor. In reality, this could be achieved either by selecting a metal with an appropriately high work function or by considerably increasing the doping level of the semiconductor (up to 10^21^ cm^−3^), which allows for lowering its potential barrier. However, in the case of p-SiC, such high doping is extremely difficult, and moreover, it lowers hole mobility and results in high ionization energies of dopants (150–200 meV) [13,131]. Furthermore, the high value of the p-type 4H-SiC work function (~7 eV) makes it difficult to find a conventional metal that can result in the formation of a low potential barrier. When manufacturing thermally stable ohmic contacts to p-SiC, the greatest attention is focused on solutions based on various compositions of Ti/Al alloys with values of ρ_c_ within the range of 10^−4–^10^−5^ Ωcm^2^ [132,133,134]. Our research team also developed the technology of successful ohmic contact formation for both n-SiC and p-SiC, which show low specific resistance (4 × 10^−5^ Ωcm^2^ and 5.8 × 10^−5^ Ωcm^2^, respectively) and good thermal stability. As a metallization for n-type 4H-SiC ohmic contacts, titanium was applied, whereas for p-type 4H-SiC, Ti/Al alloys were used [6,135].

### 6.3. Schottky Contacts

Among all technologies of semiconductor devices based on silicon carbide, the technology of the Schottky Barrier Diode is the most mature. Nevertheless, discussions and research on their fabrication methods as well as on physical phenomena occurring in these structures still continue. The interest of research groups is focused on issues related to the impact of annealing [136], substrate and interfacial defects [137,138,139], surface impurities [118], and metal thickness [140] on the junction performance. SiC-based Schottky Barrier Diodes demonstrate low reverse leakage current and relatively fast switching. Recent research efforts are aimed mostly towards a better understanding of the physical nature of the metal–semiconductor interface and, consequently, the improvement of diode performance.

At present, the commonly proposed technological solution consists of SBDs with p-type guard zones and rings (JTE—junction termination extension) that allow for increasing the breakdown voltage [141,142]. Other technological improvements include the angular ion implantation method, which has a positive effect on the process flow, reducing the damage of the wafer and thus generating cost savings, which is particularly significant from the viewpoint of mass production [143]. Simultaneously, attempts to fabricate ohmic contacts and Schottky barrier junctions favor the device miniaturization process, as in this case, the cell pitch may be smaller [137]. Optimized 4H-SiC superjunction Schottky diodes of the voltage class 1700 V were reported, the fabrication technology of which overcomes some technological challenges associated with the manufacturing of conventional superjunctions [144]. The high leakage current under high blocking voltage can be limited by the implementation of one of the specific design solutions, which include the Junction Barrier Schottky (JBS) or Trench Junction Barrier Schottky (TJBS) layout [145,146]. A SiC-merged p-i-n Schottky (MPS) layout was also proposed to withstand high surge current stress [147]. Due to the tradeoff between the basic electrical parameters, all subsequent generations of commercially available Schottky diodes benefit from the achievements resulting from the optimization of the above-mentioned structures. Also, another type of device with many possible applications has been proposed. It is a highly sensitive temperature sensor based on Ni/4H-SiC edge-terminated SBD. The highest thermal sensitivity value of such a device was 3.425 mV/K at the lowest forward current I_f_ = 10 pA [148]. However, despite the continuous interest and growing number of studies on the development of SiC-based Schottky Barrier Diode technology, the potential of these structures has not been fully exploited yet.

### 6.4. Conclusions

Although significant progress has been made in improving the processes responsible for forming metal–SiC contacts, some challenges and noteworthy issues still remain. The specific nature of the m-s structure formation is not fully understood and explained, which is why some research still continues, despite the commercialization of silicon carbide devices.

As numerous studies show, including experiments conducted by our group, some of the problems related to insufficient operation or the appearance of unexpected phenomena can be significantly alleviated by surface treatment of the semiconductor substrate between technological steps.

## 7. Power Semiconductor Assembly

Discrete SiC power components are commercially available with operating voltages in the 650 V to 1700 V range, a maximum forward current of up to 120 A, and operating temperatures as high as 175 °C [149]. However, the capabilities of SiC components are much broader; they can operate at much higher current densities (2–3 times the capability of silicon devices) and at temperatures as high as 200–300 °C.

**Figure 18:**
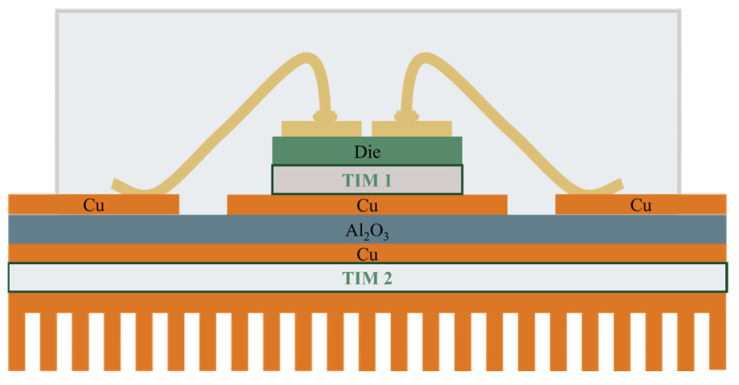
Scheme of first-generation SiC power modules. TIM1 at chip—DBC interface; TIM2 at DBC—radiator interface.

Meeting these expectations requires the development and implementation of suitable design of SiC component packages [149,150]. The first generations of SiC modules were based on solutions used in Si-based power components, as shown in Figure 18. In this solution, the top side of the SiC semiconductor is used for the electrical connections, and the bottom side is used to mount to the DBC substrate with Thermal Interface Material (TIM1) [151,152]. The electrical connections on the top side are made by wire bonding. Due to the high currents, Al wires with diameters in the range of 100–1000 µm are usually applied. Cu wires with diameters in the hundreds of microns are used in more recent devices. The use of Cu instead of Al makes it possible to increase the current-carrying capacity of connections (the electrical conductivity of Cu is 39% higher than that of Al and the thermal conductivity of Cu is 77% better than that of Al). It should be noted, however, that the use of Cu wires involves the need for shielding atmospheres during joining, which is why Cu wires coated with a thin Al layer are used. Such joints can be made using the conventional ultra-compression method at room temperature. Another solution is to use Al ribbons or Cu ribbons. Ribbons have larger cross-sections than wires, so the same connection resistance can be provided with fewer connections. It should be noted that the high inductance of wire connections (for TO enclosures in the 20–30 nH range) is a significant disadvantage for components operating at high frequencies [150].

Table 1 shows the bonding of the SiC structure to the substrate. It should have good mechanical (adhesion), electrical, and thermal properties. In the presented solution, the only way to dissipate heat is through TIM1, which fixes the structure in the housing and is responsible for cooling the SiC structures, thus limiting the maximum allowable operating temperature. It is also worth noting that the surface area for heat dissipation of the TIM1 and SiC chips is comparable.

Hence, the requirement of good thermal conductivity for the TIM1 is achieved. The same heat flux is transferred from the package to the heat sink via the TIM 2 over a much larger surface area, corresponding to the device package. What is required from TIM2 is good adhesion and acceptable thermal conductivity but also good electrical insulation. Electrical (wire bonding inductance) and thermal (single-sided cooling only) limitations forced the search for other solutions for SiC components.

**Figure 19:**
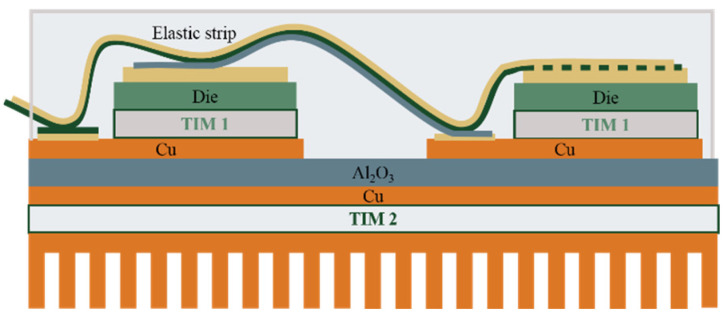
Flex printed circuit board solution (SKIN).

Other solutions have focused on reducing parasitic inductance, i.e., eliminating wire connections and replacing them with soldered ribbon connections (DLB solution—Direct Lead Bonding) or via a flex printed circuit board (SKIN solution), as shown in Figure 19 [149].

**Figure 20:**
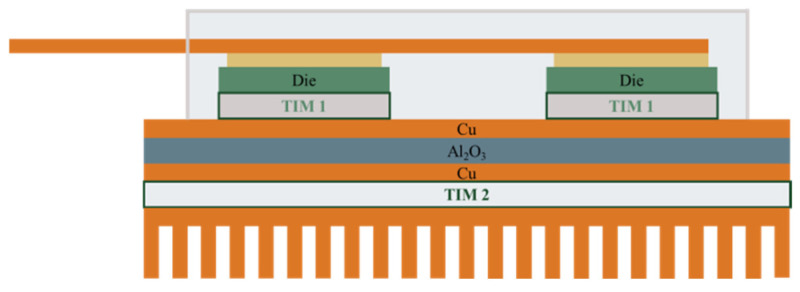
Direct Lead Bonding (DLB) structure.

When using a DLB solution, the internal inductance can be reduced to 57% of that shown in Figure 20 and internal lead resistance can be reduced to 50%, while extending reliable operation time by almost 10 times.

**Figure 21:**
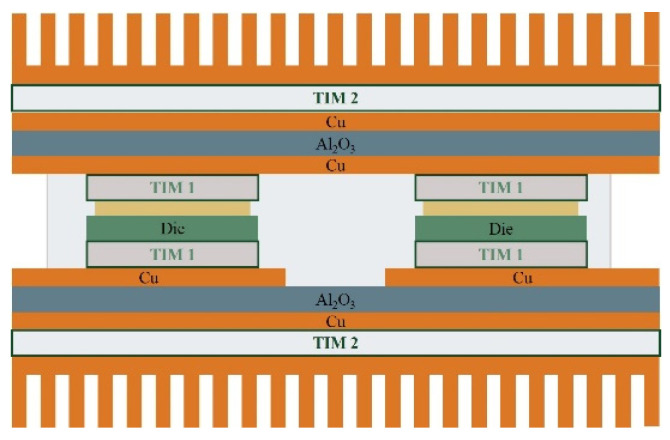
Package with double-sided cooling.

With the SKIN solution, which uses Ag paste sintering to attach a flexible PCB to the top contacts of the chip and Ag paste sintering to attach a heat sink to the DBC, a reduction in parasitic resistance of up to 10% was achieved. Nevertheless, the cooling method has not been changed in this solution; it is still single-sided. Therefore, design solutions are being sought for packages where, regardless of the elimination of wire connections, double-sided cooling is possible, as shown in Figure 21.

A much better solution is to use a double-sided package, as shown in Figure 21. In such packages, power chips are sandwiched between two ceramic substrates with Cu metallization on both sides (DBC—Direct Bonded Copper substrates). Planar electrical paths and joints can be formed in the inner Cu layers on the ceramics. The ceramic substrates provide ideal insulation between the electrical part and the outer part where bilateral cooling can be used. This symmetrical package structure, where semiconductor components are placed between the inner Cu layers, allows for flexibility in routing flat paths and placing contact pads to make joints between contact pads of active components. Joints between chip and substrate contacts are made by soldering or sintering technology based on Ag powders (TIM1 connections). This solution allows for the use of a vertical semiconductor structure in which the electrodes are arranged on both the top and bottom surfaces of the die. Compared to the traditional solution in Figure 18 with wire bonding, where all semiconductor elements are placed in a face-up position on a 2-D substrate (in the X-Y plane), the distribution of paths in the new solution allows the X-Z plane to be used more effectively. The dimension in the Z direction is usually of the order of 0.1 mm (thickness of semiconductor structures), and the paths distributed in the Cu layers on DBCs in the X or Y direction usually do not exceed 10 mm. This limits the length of joints which translates into reductions in parasitic inductance and reduced joint resistance. The sandwich design of such modules allows for heat dissipation from both sides.

Theoretically, by using double-sided cooling, the thermal resistance of a double-cooled package can be reduced relative to a single-sided package by a factor of two. Reference [150] demonstrates that the typical Rth of the single-sided cooled power module is around 0.4 Kcm^2^/W, while in the double-sided case, it is possible to reduce the Rth to less than 0.2 Kcm^2^/W. In addition, the symmetry of the package provides better mechanical restraint of the components in the housing and reduces the possibility of deformation of the package with thermal exposures, and material symmetry prevents expansion-related deformations.

### 7.1. Our Work

The work carried out by our team focused on the study of TIM1, a material and technology for the assembly of SiC chips into packages. An analysis of the literature on this topic indicates that the following assembly techniques can be used: solder die attach, sintering, SLID (Solid–Liquid Interdiffusion), or organic die attach.

Soldering has been the most widely used assembly technique. When using Pb5Sn high-lead solders, or AuSi (T_m_ = 363 °C), AuGe (T_m_ = 356 °C), or AuSn (T_m_ = 280 °C) eutectic solders, the maximum operating temperature can reach 250 °C. The thermal resistance R_thj-c_ for joints made with Pb5Sn solders is in the range of 0.65–0.67 K/W [153,154]. When the SAC-type solders are used, the maximum operating temperature must not exceed 175 °C [150]. In the case of solders, the thermal conductivity of the bonding layer is in the range of 30–50 W/mK. The research indicates that for connections made with SAC solder, R_thj-c_ in the range of 0.36–0.39 K/W was obtained.

One of the most promising and intensively studied joining technologies is sintering based on Ag [155,156,157]. The sintering technology is useful for high-temperature electronics, the joining process can be carried out at temperatures in the range of 220–300 °C, and the resulting joints are capable of working at temperatures above 500 °C. An important advantage of joints based on Ag paste sintering is a thermal conductivity of more than 150 W/mK. The joints made by Ag sintering technology were characterized by R_thj-c_ in the range of 0.82–1.58 K/W.

There are other attempts to use Solid–Liquid Interdiffusion (SLID) technology, also known as TLP (Transient Liquid-Phase Bonding). This technology is based on the formation of a bonding layer based on the intermetallic bonds between Sn or In and high-temperature-melting materials such as Au, Ag, Cu, or Ni [158,159]. The maximum operating temperatures for the Au-Sn system reached 418 °C, for the Cu-Sn system 408 °C, for the Ag-Sn system 480 °C, and for the Ag-In system 600 °C, while the joints made by SLID technology were characterized by R_thj-c_ = 0.58–1.15 K/W. In this case, the bonding layer is a film of intermetallic compounds (IMCs) whose thermal conductivity is in the range of 30–60 W/mK [160]. A comparative study showed that by using SLID technology, it is possible to obtain joints with good thermal performance at lower joining temperatures and at lower pressures.

A separate group is an organic die attach, which is a Ag paste with a small addition of resins (from several to 20% by weight) [161,162,163,164]. The addition of resins allows for pressureless sintering and for performing joining processes at temperatures below 200 °C. It is worth noting that the Ag pastes of this type, due to the presence of resins, can be used to assemble Si structures without bottom metallization. Joints formed between DBC substrate NiAu metallization and bare Si at 175 °C and 0.1 MPa have adhesion above 10 MPa and thermal resistance in the range of 0.11–0.25 K/W [165]. Table 3 compares the adhesion and thermal resistivity of Si chip joints with different metallization techniques formed by Ag pastes with 8% resin addition (TIM1) achieved in our laboratory [152,165].

Ag paste sintering technology can be applied in the assembly of SiC chips to DBA substrates (AlN ceramics with Al metallization) [166,167,168]. These connections are based on the Ag/Al interface between the SiC chip and the DBA substrate and between the DBA substrate and the heat sink. They can be manufactured using pressureless technology at temperatures in the range of 230–250 °C. They are characterized not only by efficient heat dissipation to the heat sink but also by robustness to temperature cycling [168].

### 7.2. Conclusions

The development of SiC structure assembly technology is progressing in two main directions. Firstly, it is aimed at increasing the speed of signal processing and improving cooling conditions for heavy-duty operations. This is supported by the trend towards replacing wire connections with ribbon connections, which allows for an improvement in current-carrying capacity, replacing Al wires with Cu wires covered with a thin Al layer, and further transitions to flip chip connections, i.e., direct connection of structure contacts with contact fields of DBC substrates, which enables a significant reduction in inductance. In terms of improving cooling conditions, the aim is to find solutions that allow for double-sided cooling. Secondly, new material solutions are being investigated for TIM1 (SiC–substrate interface). The usefulness of three SiC assembly technologies for substrates based on high-melting lead-free solders, SLID technologies, and Ag paste sintering was analyzed. In the case of environmentally friendly lead-free solders (AuSn or AuGe), it is necessary to use higher process temperatures (300–400 °C range), which is technologically troublesome and associated with the generation of stresses and cracks during cooling, and the solders are expensive. With SLID technology and sintering, joining temperatures are similar (220–300 °C) and operating temperatures exceeding 400 °C are achievable. The disadvantage of SLID technology is that pressure is required during joining, which is not necessary for sintering. It is worth noting that the bonding layer in Ag paste sintering technology has a very good thermal conductivity exceeding 150 W/mK, while for SLID technology, it does not exceed 50 W/mK. Here, high hopes are placed on Ag paste sintering technology. This technology is particularly interesting, as it enables not only a good bonding at the Au/Au, Ag/Al, or Ag/Ag interface but also non-metalized Si to the Au-metallized package.

## 8. Concluding Remarks

Without any doubt, SiC is a key emerging technology for the next generation of semiconductors driving the electromobility, renewable energies, smart grids, smart buildings, smart metering, and digitization of industrial processes leading to the energy transformation. SiC holds great promise for several automotive and traction applications, particularly for battery electric vehicles (BEVs) with charging systems, and has already been widely adopted. Moreover, it plays a crucial role in high-power industrial and public transport applications.

The main goal of this paper is to provide a comprehensive review of the recent advances in SiC technology. We have focused on identifying the main challenges of this technology starting with the SiC substrate growth and the defect control challenges. We describe the significant issues that are quite different from the well-established Si technology and how they have been addressed by the leading researchers in this field. We provide a comprehensive bibliography of the key advances in SiC process step development and also the most recent results from this paper’s authors.

## Figures and Tables

**Table 1:** Selected results of SiC etching using the RIE method.

Power [W]	Pressure [mTorr]	**Gases**: SF_6_/O_2_ [sccm]	SiC Etch Rate [nm/min]	SiO_2_ Etch Rate [nm/min]	Selectivity V_SiC_/V_SiO₂_
50	30	20/30	17.9	20.8	0.86
100			51.9	46.4	1.12
200			124.9	96.0	1.30
250			158.3	110.4	1.43
300			185.8	131.1	1.42
250	100		143.5	145.5	0.99
250	150		142.2	159.0	0.89

**Table 2:** Summary of the ICP-RIE results obtained for SiC structures etched with various masks.

	Al Mask	AZ4562 Photoresist
Process parameters		
SF_6_ flowrate = 100 sccm P_RIE_ = 50 W P_ICP_ = 2500 W p = 7 mTorr t = ~18.5 min	SF_6_ flowrate = 20 sccm O_2_ flowrate = 2 sccm P_RIE_ = 100 W P_ICP_ = 900 W p = 7 mTorr t = 10 min	
MESA’s height (µm)		
~8	1.3	1
Sidewall angle		
~90°	~82°	~46°

**Table 3:** Summary of adhesion and thermal resistance study for different interfaces assembled with Ag paste.

TIM Material and Interfaces	Adhesion [MPa]	Thermal Resistance [K/W]
Ag paste with interfaces Au/Au	11.5 ± 1.0	0.08 ÷ 0.14
Ag paste with interfaces Au/Si	12.6 ± 1.0	0.11 ÷ 0.26
Ag paste with SLID interfaces	12.3 ± 2.5	0.16 ÷ 0.19

## Data Availability

The data presented in this study are available upon request from the corresponding author. The data are not publicly available due to internal regulations.

## References

[1] (2022). SiC Wafer Size Trends.

[2] (2023). SiC Market Segment Size Growth Trends.

[3] Langpoklakpam C., Liu A.-C., Chu K.-H., Hsu L.-H., Lee W.-C., Chen S.-C., Sun C.-W., Shih M.-H., Lee K.-Y., Kuo H.-C. (2022). Review of Silicon Carbide Processing for Power MOSFET. Crystals.

[4] Epitaxial layer growth. Proceedings of the SOITEC, Leti Workshop.

[5] SOITEC’s Smart Cut SiC wafer manufacturing process. Proceedings of the SOITEC, Leti Workshop.

[6] Martychowiec A., Kwietniewski N., Kondracka K., Werbowy A., Sochacki M. Ti and TiAl-based ohmic contacts to 4H-SiC. Proceedings of the SPIE 11581, Photonics Applications in Astronomy, Communications, Industry, and High Energy Physics Experiments 2020, 115810W.

[7] Roccaforte F., Fiorenza P., Vivona M., Greco G., Giannazzo F. (2021). Selective Doping in Silicon Carbide Power Devices. Materials.

[8] Nipoti R., Carnera A., Alfieri G., Kranz L. (2018). About the Electrical Activation of 1 × 10^20^ cm^−3^ Ion Implanted Al in 4H-SiC at Annealing Temperatures in the Range 1500–1950 °C. Mater. Sci. Forum.

[9] Ayedh H.M., Nipoti R., Hallen A., Svensson B.G. (2015). Elimination of carbon vacancies in 4H-SiC employing thermodynamic equilibrium conditions at moderate temperatures. Appl. Phys. Lett..

[10] Defo R.K., Zhang X., Bracher D., Kim G., Hu E., Kaxiras E. (2018). Energetics and kinetics of vacancy defects in 4H-SiC. Phys. Rev. B.

[11] Laube M., Schmid F., Semmelroth K., Pensl G., Devaty R., Choyke W., Wagner G., Maier M., Choyke W.J., Matsunami H., Pensl G. (2004). Phosphorus-Related Centers in SiC. Silicon Carbide Recent Major Advances.

[12] Simonka V., Hossinger A., Selberherr S., Weinbub J. Investigation of Post-Implantation Annealing for Phosphorus-Implanted 4H-Silicon Carbide. Proceedings of the 1st International Conference on Microelectronic Devices and Technologies (MicDAT’2018).

[13] Rambach M., Bauer A.J., Ryssel H. (2008). Electrical and topographical characterization of aluminum implanted layers in 4H silicon carbide. Phys. Status Solidi B.

[14] Higashi S., Maruyama K., Hanafusa H. Activation of impurity atoms in 4H-SiC wafer by atmospheric pressure thermal plasma jet irradiation. Proceedings of the 16th International Workshop on Junction Technology (IWJT 2016).

[15] Maruyama K., Hanafusa H., Ashihara R., Hayashi S., Murakami H., Higashi S. (2015). High-efficiency impurity activation by precise control of cooling rate during atmosheric pressure thermal plasma jet annealing of 4H-SiC wafer. Jpn. J. Appl. Phys..

[16] Calabretta C., Agati M., Zimbone M., Boninelli S., Castiello A., Pecora A., Fortunato G., Calcagno L., Torrisi L., La Via F. (2019). Laser Annealing of P and Al Implanted 4H-SiC Epitaxial Layers. Materials.

[17] Zekentes K., Vasilevskiy K. (2020). Advancing Silicon Carbide Electronics Technology II Core Technologies of Silicon Carbide Device Processing.

[18] Fedeli P., Gorni M., Carnera A., Parisini A., Alfieri G., Grossner U., Nipoti R. (2016). 1950 °C Post Implantation Annealing of Al^+^ Implanted 4H-SiC: Relevance of the Annealing Time. ECS J. Solid State Sci. Technol..

[19] Canino M. (2007). Phosphorus Ion Implantation in Sic: Influence of the Annealing Conditions on Dopant Activation and Defects. Ph.D. Thesis.

[20] Sundaresan S.G., Rao M.V., Tian Y.-I., Ridgway M.C., Schreifels J.A., Kopanski J.J. (2007). Ultrahigh-temperature microwave annealing of Al^+^ - and P^+^ - implanted 4H-SiC. J. Appl. Phys..

[21] Nipoti R., Parisini A. (2019). Al^+^ Ion Implanted 4H-SiC: Electrical Activation versus Annealing Time. ECS Trans..

[22] Poggi A., Bergamini F., Nipoti R., Solmi S., Canino M., Carnera A. (2006). Effects of heating ramp rates on the characteristics of Al implanted 4H-SiC junctions. Appl. Phys. Lett..

[23] Piskorski K., Guziewicz M., Wzorek M., Dobrzański L. (2020). Investigation of Al- and N- implanted 4H-SiC applying visible and deep UV Raman scattering spectroscopy. AIP Adv..

[24] Lazar M., Laariedh F., Cremillieu P., Planson D., Leclercq J.-L. (2015). The channeling effect of Al. and N ion implantation in 4H-SiC during JFET integrated device processing. Nucl. Instrum. Methods Phys. Res. B.

[25] Nipoti R., Parisini A., Vantaggio S., Alfieri G., Carnera A., Centurioni E., Ivan E., Grossner U. (2016). 1950 °C Annealing of Al+ Implanted 4H-SiC: Sheet Resistance Dependence on the Annealing Time. Mater. Sci. Forum.

[26] Roccaforte F., Giannazzo F., Greco G. (2022). Ion Implantation Doping in Silicon Carbide and Gallium Nitride Electronic Devices. Micro.

[27] Capano M.A., Ryu S., Melloch M.R., Cooper J.A., Buss M.R. (1998). Dopant activation and surface morphology of ion implanted 4H- and 6H-silicon carbide. J. Electron. Mater..

[28] Hallen A., Linnarsson M. (2016). Ion implantation technology for silicon carbide. Surf. Coat. Technol..

[29] Rao S.P. (2005). Implant Annealing of Al Dopants in Silicon Carbide Using Silane Overpressure. Ph.D. Thesis.

[30] Jones K.A., Ervin M.H., Shah P.B., Derenge M.A., Vispute R.D., Venkatesan T., Freitas J.A. Electrical Activation Processes in Ion Implanted SiC Device Structures. Proceedings of the 17th International Conference on the Application of Accelerators in Research and Industry.

[31] Ruppalt L.B., Stafford S., Yuan D., Jones K.A., Ervin M.H., Kirchner K.W., Zheleva T.S., Wood M.C., Geil B.R., Forsythe E. (2003). Using a PLD BN/AlN composite as an annealing cap for ion implanted SiC. Solid. State Electron..

[32] Saddow S.E., Williams J.R., Isaacs-Smith T., Capano M.A., Cooper J.A., Mazzola M.S., Hsieh A.J., Casady J.B. (2000). High Temperature Implant Activation in 4H and 6H-SiC in a Silane Ambient to Reduce Step Bunching. Mater. Sci. Forum.

[33] Rao S., Bergamini F., Nipoti R., Saddow S.E. (2006). Silane overpressure post-implant annealing of Al. dopants in SiC: Cold wall CVD apparatus. Appl. Surf. Sci..

[34] Canino M., Fedeli P., Albonetti C., Nipoti R. (2020). 4H-SiC surface morphology after Al. ion implantation and annealing with C-cap, J. Microsc..

[35] Heera V., Panknin D., Skorupa W. (2001). p-Type doping of SiC by high dose Al implantation-problems and progres. Appl. Surf. Sci..

[36] Pensl G., Schmid F., Ciobanu F., Laube M., Reshanov S.A., Schulze N., Semmelroth K., Schöner A., Wagner G., Nagasawa H. (2003). Electrical and Optical Characterization of SiC. Mater. Sci. Forum.

[37] Asada S., Okusa T., Kimoto T., Suda J. (2016). Hall scattering factors in p-type 4H-SiC with various doping concentrations. Appl. Phys. Express.

[38] Matsuura H., Geelvinck H., Reynst S. (2011). Determination methods of densities and energy levels of impurities and defects affecting majority-carrier concentration in next-generation semiconductors. Advances in Condensed Matter and Materials Research.

[39] Kimoto T., Cooper J.A. (2014). Device Processing of Silicon Carbide. Fundamentals of Silicon Carbide Technology: Growth, Characterization, Devices and Applications.

[40] Darmody C., Goldsman N. (2019). Incomplete ionization in aluminum-doped 4H-silicon carbide. J. Appl. Phys..

[41] Hidaka A., Kondo Y., Takeshita A., Matsuura H., Eto K., Ji S., Kojima K., Kato T., Yoshida S., Okumura H. (2023). Comparison of temperature-dependent resistivity of heavily Al- and N-codoped 4H-SiC grown by physical vapor transport and heavily Al-doped 4H-SiC grown by chemical vapor deposition. Jpn. J. Appl. Phys..

[42] Matsuura H., Hidaka A., Ji S., Eto K., Ishida Y., Yoshida S. (2023). Negative Hall coefficient in band conduction region in heavily Al-doped 4H-SiC. J. Appl. Phys..

[43] Mletsching K., Rommel M., Pobegen G., Schustereder W., Pichler P. (2022). Aluminum Activation in 4H-SiC Measured on Laterally Contacted MOS Capacitors with a Burried Current-Spreading Layer. Mater. Sci. Forum.

[44] Kato M., Di J., Ohkouchi Y., Mizuno T., Ichimura M., Kojima K. (2022). Hole capture cross section of the Al acceptor level in 4H-SiC. Mater. Today Commun..

[45] Stengl R., Gosele U., Fellinger C., Beyer M., Walesch S. (1986). Variation of lateral doping as a field terminator for high-voltage power devices. IEEE Trans. Electron. Devices.

[46] Baliga B.J. (2006). Silicon Carbide Power Devices.

[47] Taube A., Sochacki M. (2020). Edge termination design for 1.7 kV silicon carbide p-i-n diodes. Bull. Pol. Acad. Tech..

[48] Deng X., Li L., Wu J., Li C., Chen W., Li J., Li Z., Zhang B. (2017). A Multiple-Ring-Modulated JTE Technique for 4H-SiC Power Device with Improved JTE-Dose Window. IEEE Trans. Electron. Devices.

[49] Li X.Q., Tone K., Cao L.H., Alexandrov P., Fursin L., Zhao J.H. (2000). Theoretical and Experimental Study of 4H-SiC Junction Edge Termination. Mater. Sci. Forum.

[50] Sung W., Baliga B.J. (2017). A Comparative Study 4500-V Edge Termination Techniques for SiC Devices. IEEE Trans. Electron Devices.

[51] Yuan H., Song Q., Tang X., Yuan L., Yang S., Tang G., Zhang Y., Zhang Y. (2016). Trench Multiple Floating Limiting Rings Termination for 4H-SiC High-Voltage Devices. IEEE Electron. Device Lett..

[52] Niwa H., Feng G., Suda J., Kimoto T. (2012). Breakdown Characteristics of 15-kV-Class 4H-SiC PiN Diodes with Various Junction Termination Structures. IEEE Trans. Electron. Devices.

[53] Feng G., Suda J., Kimoto T. (2012). Space-Modulated Junction Termination Extension for Ultrahigh-Voltage p-i-n Diodes in 4H-SiC. IEEE Trans. Electron. Devices.

[54] Ghandi R., Buono B., Domeij M., Malm G., Zetterling C.-M., Ostling M. (2009). High-Voltage 4H-SiC PiN Diodes with Etched Junction Termination Extension. IEEE Electron. Device Lett..

[55] Wen Y., Xu X.-J., Tao M.-L., Lu X.-F., Deng X.-C., Li X., Li J.-T., Li Z.-Q., Zhang B. (2020). Characterization and Fabrication of the CFM-JTE for 4H-SiC Power Device with High-Efficiency Protection and Increased JTE Dose Tolerance Window. Nanoscale Res. Lett..

[56] Huff M. (2021). Recent Advances in Reactive Ion Etching and Applications of High-Aspect-Ratio Microfabrication. Micromachines.

[57] Szczęsny A., Śniecikowski P., Szmidt J., Werbowy A. (2003). Reactive ion etching of novel materials—GaN and SiC. Vacuum.

[58] Casady J.B., Luckowski E.D., Bozack M., Sheridan D., Johnson R.W., Williams J.R. (1996). Etching of 6H-SiC and 4H-SiC using NF_3_ in a Reactive Ion Etching System. J. Electrochem. Soc..

[59] Seok O., Kim Y.-J., Bahng W. (2020). Micro-trench free 4H-SiC etching with improved SiC/SiO_2_ selectivity using inductively coupled SF_6_/O_2_/Ar plasma. Phys. Scr..

[60] Lebedeva N.M., Samsonova T.P., Il’inskaya N.D., Troshkov S.I., Ivanov P.A. (2020). Formation of SiC Mesastructures with Gently Sloping Sidewalls by Dry Selective Etching through a Photoresist Mask. Solid. State Electron..

[61] Pearton S.J., Cheung R. (2006). Dry etching of SiC. Silicon Carbide Micromechanical Systems for Harsh Environments.

[62] Jiang L., Plank N.O.V., Blauw M.A., Cheung R., van der Drift E. (2004). Dry etching of SiC in inductively coupled Cl_2_/Ar plasma. J. Phys. D Appl. Phys..

[63] Wang J.J., Lambers E.S., Pearton S.J., Ostling M., Zetterling C.-M., Grow J.M., Ren F. (1997). ICP Etching of SiC. MRS Online Proc. Libr..

[64] Liu R., Wu H., Zhang H., Li C., Tian L., Li L., Li J., Wu J., Pan P. (2020). A dry etching method for 4H-SiC via using photoresist mask. J. Cryst. Growth.

[65] Biscarrat J., Michaud J.-F., Collard E., Alquier D. (2013). ICP etching of 4H-SiC substrates. Mater. Sci. Forum.

[66] Yih P.H., Saxena V., Steckl A.J. (2001). A Review of SiC Reactive Ion Etching in Fluorinated Plasmas. Phys. Status Solidi B.

[67] Sung H.-K., Qiang T., Yao Z., Li Y., Wu Q., Lee H.-K., Park B.-D., Lim W.-S., Park K.-H., Wang C. (2017). Vertical and bevel-structured SiC etching techniques incorporating different gas mixture plasmas for various microelectronic applications. Sci. Rep..

[68] Racka-Szmidt K., Stonio B., Żelazko J., Filipiak M., Sochacki M. (2022). A Review: Inductively Coupled Plasma Reactive Ion Etching of Silicon Carbide. Materials.

[69] Tanaka S., Rajanna K., Abe T., Esashi M. (2001). Deep reactive ion etching of silicon carbide. J. Vac. Sci. Tech. B.

[70] Luna L.E., Tadjer M.J., Anderson T.J., Imhoff E.A., Hobart K.D., Kub F.J. (2017). Deep reactive ion etching of 4H-SiC via cyclic SF_6_/O_2_ segments. J. Micromech. Microeng..

[71] Donnelly V.M., Kornblit A. (2013). Plasma etching: Yesterday, today, and tomorrow. J. Vac. Sci. Technol. A.

[72] Davis R.F. (2017). Silicon Carbide. Reference Module in Materials Science and Materials Engineering.

[73] Afanasev V.V., Bassler M., Pensl G., Schulz M. (1997). Intrinsic SiC/SiO_2_ Interface States. Phys. Status Solidi A Appl. Res..

[74] Berens J., Rasinger F., Aichinger T., Heuken M., Krieger M., Pobegen G. (2019). Detection and Cryogenic Characterization of Defects at the SiO_2_/4H-SiC Interface in Trench MOSFET. IEEE Trans. Electron. Devices.

[75] Roccaforte F., Fiorenza P., Greco G., Lo Nigro R., Giannazzo F., Iucolano F., Saggio M. (2018). Emerging trends in wide band gap semiconductors (SiC and GaN) technology for power devices. Microelectron. Eng..

[76] Tachiki K., Kaneko M., Kimoto T. (2021). Mobility improvement of 4H-SiC (0001) MOSFETs by a three-step process of H_2_ etching, SiO_2_ deposition, and interface nitridation. Appl. Phys. Express.

[77] Fujita E., Sometani M., Hatakeyama T., Harada S., Yano H., Hosoi T., Shimura T., Watanabe H. (2018). Insight into enhanced field-effect mobility of 4H-SiC MOSFET with Ba incorporation studied by Hall effect measurements. AIP Adv..

[78] Mingues C., Charitat G. Efficiency of junction termination techniques vs. oxide trapped charges. Proceedings of the 9th International Symposium on Power Semiconductor Devices and IC’s.

[79] Kang I.H., Na M.K., Seok O., Moon J.H., Kim H.W., Kim S.C., Bahng W., Kim N.K., Park H.-C., Yang C.H. (2017). Effect of surface passivation on breakdown voltages of 4H-SiC Schottky barrier diodes. J. Korean Phys. Soc..

[80] Akiyama T., Ito A., Nakamura K., Ito T., Kageshima H., Uematsu M., Shiraishi K. (2015). First-principles investigations for oxidation reaction processes at 4H-SiC/SiO_2_ interface and its orientation dependence. Surf. Sci..

[81] Shen X., Tuttle B.R., Pantelides S.T. (2013). Competing atomic and molecular mechanisms of thermal oxidation—SiC versus Si. J. Appl. Phys..

[82] Knaup J.M., Deak P., Frauenheim T., Gali A., Hajnal Z., Choyke W.J. (2005). Theoretical study of the mechanism of dry oxidation of 4H-SiC. Phys. Rev. B Condens. Matter.

[83] Bockstedte M., Mattausch A., Pankratov O. (2004). Ab initio study of the annealing of vacancies and interstitials in cubic SiC: Vacancy-interstitial recombination and aggregation of carbon interstitials. Phys. Rev. B Condens. Matter.

[84] Deak P., Knaup J.M., Thill C., Frauenheim T., Gali A. (2008). The mechanism of defect creation and passivation at the SiC/SiO_2_ interface. J. Phys. D Appl. Phys..

[85] Knaup J., Deak P., Frauenheim T., Gali A., Hajnal Z., Choyke W.J. (2005). Defects in SiO_2_ as the possible origin of near interface traps in the SiC/SiO_2_ system: A systematic theoretical study. Phys. Rev. B Condens. Matter.

[86] Wang S., Dhar S., Wang S.-R., Ahyi A.C., Franceschetti A., Williams J.R., Feldman L.C., Pantelides S.T. (2007). Bonding at the SiC-SiO_2_ interface and the effects of nitrogen and hydrogen. Phys. Rev. Lett..

[87] Kaneko T., Tajima N., Yamasaki T., Nara J., Schimizu T., Kato K., Ohno T. (2018). Hybrid density functional analysis of distribution of carbon-related defect levels at 4H-SiC(0001)/SiO_2_ interface. Appl. Phys. Express.

[88] Tajima N., Kaneko T., Yamasaki T., Nara J., Schimizu T., Kato K., Ohno T. (2018). First-principles study on C = C defects near SiC/SiO_2_ interface: Defect passivation by double-bond saturation. Jpn. J. Appl. Phys..

[89] Afanas’ev V.V., Bassler M., Pensl G., Schulz M.J., Stein von Kamienski E. (1996). Band offsets and electronic structure of SiC/SiO_2_ interfaces. J. Appl. Phys..

[90] Kobayashi H., Sakurai T., Takahashi M., Nishioka Y. (2003). Interface states at SiO_2_/6H-SiC (0001) interfaces observed by x-ray photoelectron spectroscopy measurements under bias: Comparison between dry and wet oxidation. Phys. Rev. B Condens. Matter.

[91] Dalibor T., Pensl G., Matsunami H., Kimoto T., Choyke W.J., Schöner A., Nordell N. (1997). Deep Defect Centres in Silicon Carbide Monitored with Deep Level Transient Spectroscopy. Phys. Status Solidi A Appl. Res..

[92] Wang Z., Zhang Z., Liu S., Shao C., Robertson J., Guo Y. (2022). Impact of carbon-carbon defects at the SiO_2_/4H-SiC (0001) interface: A first-principles calculation. J. Phys. D Appl. Phys..

[93] Matsushita Y., Oshiyama A. (2018). Structural stability and energy levels of carbon-related defects in amorphous SiO_2_ and its interface with SiC. Jpn. J. Appl. Phys..

[94] Salemi S., Goldsman N., Akturk A., Lelis A. Density Functional Theory Based Investigation of Defects and Passivation of 4H-Silicon Carbide/SiO_2_ Interfaces. Proceedings of the International Conference on Simulation of Semiconductor Processes and Devices (SISPAD 2012).

[95] Tachiki K., Kimoto T. (2001). Improvement of Both n-and p-Channel Mobilities in 4H-SiC MOSFETs by High-Temperature N_2_ Annealing. IEEE Trans. Electron. Devices.

[96] Król K., Kalisz M., Sochacki M., Szmidt J. (2013). The Influence of Post-Oxidation Annealing Process in O_2_ and N_2_O on the Quality of Al/SiO_2_/n-Type 4H-SiC MOS Interface. Mater. Sci. Forum.

[97] Król K., Sochacki M., Turek M., Żuk J., Przewlocki H.M., Gutt T., Borowicz P., Guziewicz M., Szuber J., Kwoka M. (2013). Influence of Nitrogen Implantation on Electrical Properties of Al/SiO_2_/4H-SiC MOS Structure. Mater. Sci. Forum.

[98] Okamoto D., Yano H., Hatayama T., Fuyuki T. (2010). Removal of near-interface traps at SiO_2_/4H-SiC (0001) interfaces by phosphorus incorporation. Appl. Phys. Lett..

[99] Krol K., Sochacki M., Turek M., Żuk J., Borowicz P., Teklińska D., Konarski P., Miśnik M., Domanowska A., Michalewicz A. (2015). Influence of Phosphorus Implantation on Electrical Properties of Al/SiO_2_/4H-SiC MOS Structure. Mater. Sci. Forum.

[100] Okamoto D., Yano H., Hirata K., Hatayama T., Fuyuki T. (2010). Improved Inversion Channel Mobility in 4H-SiC MOSFETs on Si Face Utilizing Phosphorus-Doped Gate Oxide. IEEE Electron. Device Lett..

[101] Król K., Sochacki M., Strupinski W., Racka K., Guziewicz M., Konarski P., Misnik M., Szmidt J. (2015). Chlorine-enhanced thermal oxides growth and significant trap density reduction at SiO_2_/SiC interface by incorporation of phosphorus. Thin Solid Films.

[102] Okamoto D., Sometani M., Harada S., Kosugi R., Yonezawa Y., Yano H. (2014). Improved Channel Mobility in 4H-SiC MOSFETs by Boron Passivation. IEEE Electron. Device Lett..

[103] Tuttle B.R., Dhar S., Ryu S.-H., Zhu X., Williams J.R., Feldman L.C., Pantelides S.T. (2011). High electron mobility due to sodium ions in the gate oxide of SiC-metal-oxide-semiconductor field-effect-transistors. J. Appl. Phys..

[104] Lichtenwalner D.J., Cheng L., Dhar S., Agarwal A., Palmour J.W. (2014). High mobility 4H-SiC (0001) transistors using alkali and alkaline earth interface layer. Appl. Phys. Lett..

[105] Berens J., Bichelmaier S., Fernando N.K., Thakur P.K., Lee T.-L., Mascheck M., Wiell T., Eriksson S.K., Kahk J.M., Lischner J. (2020). Effects of nitridation on SiC/SiO_2_ structures studies by hard X-ray photoelectron spectroscopy. J. Phys. Energy.

[106] Brzozowski E., Kaminski M., Taube A., Sadowski O., Krol K., Guziewicz M. (2023). Carrier Trap Density Reduction at SiO_2_/4H-Silicon Carbide Interface with Annealing Process in Phosphoryl Chloride and Nitride Oxide Atmospheres. Materials.

[107] Gavrikov A., Knizhnik A., Safonov A., Scherbinin A., Bagutur’yants A., Potapkin B., Chatterjee A., Matocha K. (2008). First-principles-based investigation of kinetic mechanism of SiC(0001) dry oxidation including defect generation and passivation. J. Appl. Phys..

[108] Zhang Y.-J., Yin Z.-P., Su Y., Wang D.-J. (2018). Passivation of carbon dimer defects in amorphous SiO_2_/4H-SiC (0001) interface: A first-principles study. Chin. Phys. B.

[109] Wang Z., Zhang Z., Shao C., Robertson J., Liu S., Guo Y. (2020). Defects and Passivation Mechanism of the Suboxide Layers at SiO_2_/4H-SiC (0001) Interface: A First-Principles Calculation. IEEE Trans. Electron. Devices.

[110] Sharma Y.K., Ahyi A.C., Issacs-Smith T., Shen X., Pantelides S.T., Zhu X., Feldman L.C., Rozen J., Williams J.R. (2012). Phosphorus passivation of the SiO_2_/4H-SiC interface. Solid. State Electron..

[111] Xu Y., Xu C., Liu G., Lee H.D., Shubeita S.M., Jiao C., Modic A., Ahyi A.C., Sharma Y., Wan A. (2015). Concentration, chemical bonding, and etching behavior of P and N at the SiO_2_/SiC (0001) interface. J. Appl. Phys..

[112] Kobayashi T., Kimoto T. (2017). Carbon ejection from a SiO_2_/SiC(0001) interface by annealing in high-purity Ar. Appl. Phys. Lett..

[113] Jayawardena A., Shen X., Mooney P.M., Dhar S. (2018). Mechanism of phosphorus passivation of near-interface oxide traps in 4H-SiC MOS devices investigated by CCDLTS and DFT calculation. Semicond. Sci. Technol..

[114] Jiao C., Ahyi A.C., Xu C., Morisette D., Feldman L.C., Dhar S. (2016). Phosho-silicate glass gated 4H-SiC metal-oxide-semiconductor devices: Phosphorus concentration dependence. J. Appl. Phys..

[115] Kobayashi T., Okuda T., Tachiki K., Ito K., Matsushita Y., Kimoto T. (2020). Design and formation of SiC (0001)/SiO_2_ interfaces via Si deposition followed by low-temperature oxidation and high-temperature nitridation. Appl. Phys. Express.

[116] Pascu R., Romanitan C., Varasteanu P., Kusko M.A. (2019). Reliable Technology for Advanced SiC-MOS Devices Based on Fabrication of High Quality Silicon Oxide Layers by Converting a-Si. IEEE J. Electron. Devi..

[117] Simonka V., Hössinger A., Weinbub J., Selberherr S. (2016). Growth rates of dry thermal oxidation of 4H-silicon carbide. J. Appl. Phys..

[118] Kwietniewski N., Sochacki M., Szmidt J., Guziewicz M., Kaminska E., Piotrowska A. (2008). Influence of surface cleaning effects on properties of Schottky diodes on 4H—SiC. Appl. Surf. Sci..

[119] Monnoye S., Turover D., Vicente P., Choyke W.J., Matsunami H., Pensl G. (2004). Surface Preparation Techniques for SiC Wafers. Silicon Carbide Recent Major Advances.

[120] Anzalone R., Piluso N., Salanitri M., Lorenti S., Arena G., Coffa S. (2017). Hydrogen etching influence on 4H-SiC homo-epitaxial layer for high power device. Mater. Sci. Forum.

[121] Watanabe H., Ohmi H., Kakiuchi H., Hosoi T., Shimura T., Yasutake K. (2011). Surface cleaning and etching of 4H-SiC(0001) using high-density atmospheric pressure hydrogen plasma. J. Nanosci. Nanotechnol..

[122] Gale G.W., Cui H., Reinhardt K.A., Reinhardt K.A., Kern W. (2018). Aqueous Cleaning and Surface Conditioning Processes. Handbook of Silicon Wafer Cleaning Technology.

[123] Huang Y., Buettner J., Lechner B., Wachutka G. (2019). The impact of non-ideal ohmic contacts on the performance of high-voltage SIC MPS diodes. Mater. Sci. Forum.

[124] Roccaforte F., Vivona M., Greco G., Lo Nigro R., Giannazzo F., Di Dranco S., Bongiorno C., Iucolano F., Frazzetto A., Rascuna S. (2017). Ti/Al-based contacts to p-type SiC and GaN for power device applications. Phys. Status Solidi A.

[125] Rao S., Pangallo G., Della Corte F.G. (2015). Highly Linear Temperature Sensor Based on 4H-Silicon Carbide p-i-n Diodes. IEEE Electron. Device Lett..

[126] Lanni L., Malm B.G., Ostling M., Zetterling C.-M. (2013). 500 °C bipolar integrated OR/NOR Gate in 4H-SiC. IEEE Electron. Device Lett..

[127] Sung W., Baliga B.J. (2016). Monolithically Integrated 4H-SiC MOSFET and JBS Diode (JBSFET) Using a Single Ohmic/Schottky Process Scheme. IEEE Electron. Device Lett..

[128] Han C., Zhang Y., Song Q., Zhang Y., Tang X., Yang F., Niu Y. (2015). An Improved ICP Etching for Mesa-Terminated 4H-SiC p-i-n Diodes. IEEE Trans. Electron. Devices.

[129] Vivona M., Greco G., Bongiorno C., Lo Nigro R., Scalese S., Roccaforte F. (2017). Electrical and structural properties of surfaces and interfaces in Ti/Al/Ni Ohmic contacts to p-type implanted 4H-SiC. Appl. Surf. Sci..

[130] Roccaforte F., Vivona M., Greco G., Lo Nigro R., Giannazzo F., Rascuna S., Saggio M. (2018). Metal/semiconductor contacts to silicon carbide: Physics and technology. Mater. Sci. Forum.

[131] Roccaforte F., Giannazzo F., Raineri V. (2010). Nanoscale transport properties at silicon carbide interfaces. J. Phys. D Appl. Phys..

[132] Zhang Y., Guo T., Tang X., Yang J., He Y., Zhang Y. (2018). Thermal stability study of n-type and p-type ohmic contacts simultaneously formed on 4H-SiC. J. Alloys Compd..

[133] Abi-Tannous T., Soueidan M., Ferro G., Lazar M., Raynaud C., Toury B., Beaufort M.-F., Barbot J.-F., Dezellus O., Planson D. (2016). A Study on the Temperature of Ohmic Contact to p-Type SiC Based on Ti3SiC2 Phase. IEEE Trans. Electron. Devices.

[134] Nicholls J.R., Dimitrijev S. (2020). A compact model for sic schottky barrier diodes based on the fundamental current mechanisms. IEEE J. Electron. Devi..

[135] Mysliwiec M., Sochacki M., Kisiel R., Guziewicz M., Wzorek M. TiAl-based ohmic contacts on p-type SiC. Proceedings of the 34th International Spring Seminar on Electronics Technology (ISSE).

[136] Xu H., Ren N., Wu J., Zhu Z., Guo Q., Sheng K. (2021). The Impact of Process Conditions on Surge Current Capability of 1.2 kV SiC JBS and MPS diodes. Materials.

[137] Shimizu H., Watanabe N., Morikawa T., Shima A., Iwamuro N. (2020). 1.2 kV silicon carbide Schottky barrier diode embedded MOSFETs with extension structure and titanium-based single contact. Jpn. J. Appl. Phys..

[138] Hayashi S., Yamasshita T., Miyazato M., Niyajima M., Senzaki J., Kato T., Yonezawa Y., Kojima K., Okamura H. (2019). Structural analysis of interfacial dislocations and expanded single Shockley-type stacking faults in forward-current degradation of 4H-SiC p-i-n diodes. Jpn. J. Appl. Phys..

[139] Kumar V., Maan A.S., Akhtar J. (2020). Electronic transport in epitaxial 4H–SiC based Schottky diodes modified selectively by swift heavy ions. Mater. Sci. Semicond. Process..

[140] Bellocchi G., Vivona M., Bongiorno C., Badala P., Bassi A., Rascuna S., Roccaforte F. (2021). Barrier height tuning in Ti/4H-SiC Schottky diodes. Solid. State. Electron..

[141] Basov M. (2021). Schottky diode temperature sensor for pressure sensor. Sens. Actuators A Phys..

[142] Nakayama K., Masuda S., Satoh N., Yamamoto H. (2020). Evaluation of silicon carbide Schottky barrier diode within guard ring by multifunctional scanning probe microscopy. Jpn. J. Appl. Phys..

[143] Jiang C., Wu H., Tian L., Li J., Liu R., Zhu T., Li L., Zhang H., Jiao Q., Wu B. (2020). Angular rotation ion implantation technology in SiC for 4H-SiC junction barrier Schottky rectifiers. J. Cryst. Growth.

[144] Baker G.W.C., Chan C., Renz A.B., Qi Y., Dai T., Li F., Shah V.A., Mawby P.A., Antoniou M., Gammon P.M. (2021). Optimization of 1700-V 4H-SiC Superjunction Schottky Rectifiers with Implanted P-Pillars for Practical Realization. IEEE Trans. Electron Devices.

[145] Zhu L., Chow T.P. (2008). Advanced high-voltage 4H-SiC Schottky rectifiers. IEEE Trans. Electron. Devices.

[146] Ren N., Wang J., Sheng K. (2014). Design and experimental study of 4H-SiC trenched junction barrier Schottky diodes. IEEE Trans. Electron. Devices.

[147] Sawant S., Baliga B.J. A comparative study of high voltage (4 kV) power rectifiers PiN/MPS/SSD/SPEED. Proceedings of the 11th International Symposium on Power Semiconductor Devices and ICs (ISPSD’99).

[148] Kumar V., Verma J., Maan A.S., Akhtar J. (2020). Epitaxial 4H–SiC based Schottky diode temperature sensors in ultra-low current range. Vacuum.

[149] Chen C., Luo F., Kang Y. (2017). A review of SiC power module packaging: Layout, material system and integration. CPSS Trans. Power Electron..

[150] Liu M., Coppola A., Alvi M., Anwar M. (2022). Comprehensive Review and State of Development of Double-Sided Cooled Package Technology for Automotive Power Modules. IEEE Open J. Power Electron..

[151] Myśliwiec M., Kisiel R., Guziewicz M. (2015). Material and technological aspects of high-temperature SiC device packages reliability. Microelecronics Int..

[152] Kisiel R., Śpiewak P., Kruszewski M.J. Ag-based Thermal Interface Materials for GaN-on-Si Assembly Chips in Power Applications. Proceedings of the 44th International Spring Seminar on Electronics Technology (ISSE).

[153] Kim D., Nagao S., Chen C., Wakasugi N., Yamamoto Y., Suetake A., Takemasa T., Suganuma K. (2020). Online Thermal Resistance and Reliability Characteristic Monitoring of Power Modules with Ag Sinter Joining and Pb, Pb-Free Solders During Power Cycling Test by SiC TEG Chip. IEEE Trans. Power Electron..

[154] Pei L.S., Pan B., Zhang H., Ng W., Wu B., Siow K.S., Sabne S., Tsuriya M. High temperature Pb-free die attach material project phase 1: Survey result. Proceedings of the International Conference on Electronics Packaging (ICEP).

[155] Zhao W., Yao Q. An Overview of the assembly and packaging of wide band gap semiconductor technologies. Proceedings of the 18th International Conference on Electronic Packaging Technology (ICEPT).

[156] Fenech M., Siebenhuhnner M., Durham J., Susanti L., Khaselev O., Boureghda M., Joguet J., Wu W., Dutt G. Power Package Attach by Silver Sintering—Process, Performance & Reliability. Proceedings of the PCIM Asia 2020 International Exhibition and Conference for Power Electronics, Intelligent Motion, Renewable Energy and Energy Management.

[157] Yeom J., Li C.-F., Suganama K. Sintering mechanism of micron/submicron-size silver particles. Proceedings of the International Conference on Electronics Packaging and IMAPS All Asia Conference (ICEP-IAAC).

[158] Aasmundtveit K.E., Jiang H., Tollefsen T.A., Luu T.-T., Nguyen H.-V. Phase Determination in SLID Bonding. Proceedings of the 7th Electronic System-Integration Technology Conference (ESTC).

[159] Larsson A., Tollefsen T.A., Aasmundtveit K.E., Lovvik O.M. Liquid Solid Diffusion (LSD) bonding: A novel joining technology. Proceedings of the 21st European Microelectronics and Packaging Conference (EMPC) & Exhibition.

[160] Hou F., Sun Z., Su M., Fan J., You X., Li J., Wang Q., Cao L., Zhang G. (2024). Review of Die-Attach Materials for SiC High-Temperature Packaging. IEEE Trans. Power Electron..

[161] Zhang Z., Chen C., Suetake A., Hsieh M.-C., Iwaki A., Suganuma K. (2021). Pressureless and low-temperature sinter-joining on bare Si, SiC and GaN by a Ag flake paste. Scr. Mater..

[162] Wang M., Mei Y.-H., Jin J., Chen S., Li X., Lu G.-Q. (2021). Pressureless Sintered Silver Die-Attach at 180^o^C for Power Electronics Packaging. IEEE Trans. Power Electron..

[163] Fan J., Xu D., Zhang H., Qian C., Fan X., Zhang G. (2020). Experimental Investigation on the Sintering Kinetics of Nanosilver Particles Used in High-Power Electronic Packaging. IEEE Trans. Compon. Packag. Manuf. Technol..

[164] Zhang X., Zhang Y., Wang C., Zhu P., Xiang B., Zhao T., Xu L., Sun R. Exploration of Key Factors for the Sintering of Micro-Nano Silver Paste. Proceedings of the 23rd International Conference on Electronic Packaging Technology (ICEPT).

[165] Kisiel R., Mysliwiec M. Combination of Solid-Liquid Interdiffusion and Sintering Bonding for GaN Devices. Proceedings of the 40th International Spring Seminar on Electronics Technology (ISSE).

[166] Chen C., Kim D., Zhang Z., Wakasugi N., Liu Y., Hsieh M.-C., Zhao S., Sustake A., Suganuma K. (2022). Interface-Mechanical and Thermal Characteristics of Ag Sinter Joining on Bare DBA Substrate During Aging, Thermal Shock and 1200 W/cm^2^ Power Cycling Tests. IEEE Trans. Power Electron..

[167] Myśliwiec M., Pavlov K., Kisiel R. Silver sintering Application in Packaging Al Metallized Chip onto Metallized Substrates. Proceedings of the 47th International Spring Seminar on Electronics Technology (ISSE).

[168] Chen C., Suetake A., Huo F., Kim D., Zhang Z., Hsieh M.-C., Li W., Wakasugi N., Takeshita K., Yamaguchi Y. (2024). Development of SiC Power Module Structure by Micron-Sized Ag-Paste Sinter Joining on Both Die and Heatsink to Low-Thermal-Resistance and Superior Power Cycling Reliability. IEEE Trans. Power Electron..

